# Clonal biases dictate availability of colonic cancer driver mutations for transformation

**DOI:** 10.1038/s41467-026-71944-5

**Published:** 2026-04-16

**Authors:** Nefeli Skoufou-Papoutsaki, Richard Kemp, Sam Adler, Kate Marks, Anne-Claire Girard, Shenay Mehmed, Filipe C. Lourenço, Elisa B. Moutin, Rogier Ten Hoopen, Edward Morrissey, Douglas J. Winton, David S. Tourigny

**Affiliations:** 1 CRUK Cambridge Institute, University of Cambridge, Cambridge, UK; 2 Wellcome-MRC Cambridge Stem Cell Institute, University of Cambridge, Cambridge, UK; 3 Pathology and Data Analytics, Leeds Institute of Medical Research, University of Leeds School of Medicine, University of Leeds, Leeds, UK; 4 Department of Oncology, University of Cambridge, Cambridge, UK; 5 MRC Weatherall Institute of Molecular Medicine, John Radcliffe Hospital, University of Oxford, Oxford, UK; 6 School of Mathematics, University of Birmingham, Birmingham, UK

**Keywords:** Cancer genetics, Ageing

## Abstract

Aged normal tissues harbour cancer mutations predisposing to transformation. However, how different pro-oncogenic events in the human colon compare in frequency, behaviour and subsequent transformation risk remains unclear. Here, we analyse mutation hotspot regions in five colorectal cancer genes (*APC*, *KRAS*, *TP53*, *FBXW7* and *CTNNB1*) using targeted sequencing of 76,800 normal colonic glands from 56 patients. We show that cancer-driving mutations are present in all genes in histologically normal tissue. Reconstruction of clone dynamics reveals that *FBXW7* R465C mutations preferentially become fixed within the tissue, whereas *KRAS* G12 mutations strongly promote expansion. Modelling mutation order indicates that early loss of both *APC* copies increasingly favours an *APC*-first pathway with age, while *KRAS* activation is equally likely to initiate events in younger individuals. Spatial transcriptomics highlights phenotypic heterogeneity among *KRAS* mutant clones, with mixed lineage presentation observed only in a subset, a state linked to elevated transformation risk in other organs.

## Abstract (web summary)

Currently, little is known about the baseline burden or the clonal dynamics of cancer driver mutations in the normal human colon. Here, the authors employ targeted amplicon sequencing, computational modeling and spatial transcriptomics to characterize the prevalence, behavior and evolutionary fate of cancer driver mutant clones in the normal human colon, allowing a reassessment of the origins of colorectal cancer.

## Introduction

The status of cancer driver mutations in normal tissue, specifically their prevalence, selective advantage and clonal behaviour, is fundamental to understanding cancer aetiology, because the routes available for transformation depend on which driver mutations exist and how they co-occur and interact during early evolution. Despite increasing evidence of somatic evolution in normal tissues relatively little is known about the baseline burden of cancer driver mutations in the normal human colonic epithelium. Lee-Six et al.^1^ reported that mutational burden in the colon is dominated by ageing-associated processes and estimated that ~1% of crypts in 50–60-year-olds contain at least one cancer driver mutation,although recurrent mutations in canonical colorectal cancer (CRC) genes, such as *APC* and *KRAS,* were not detected. In contrast targeted studies by us and others have revealed recurrent *KRAS* G12 driver mutations in normal colon and demonstrated that positive selection strongly influences the cumulative burden of *KRAS*-mutant clones by promoting biased clonal expansion^2–4^. However, it remains unclear whether other CRC driver genes undergo selection in normal colon, and what consequences this has for the stepwise acquisition of mutations and the ordering of transformative events in colorectal carcinogenesis.

Recent evolutionary models of CRC, including the Big Bang model of tumour growth^5^, propose that many key driver events occur early, followed by rapid clonal expansions that establish much of the intra-tumoural architecture before clinical detection. This model implies that the earliest stages of CRC evolution must already involve multiple driver hits and recent estimates suggest between three and five mutational events are required for malignant transformation^6–9^. Understanding which of these events are already present in the normal colon prior to any detectible histological or morphological changes to the tissue, is therefore essential to reconstruct the origins of CRC and to distinguish neutral clonal drift from selection-driven evolution. Identifying early events in somatic evolution has particular relevance for early-onset CRC, which arises decades earlier than expected^10^ yet typically does not show evidence of grossly elevated mutational burden^11^. Instead early-onset CRC might emerge through alternative evolutionary routes compared with late-onset disease^12^. Moreover, the molecular phenotypes of driver-mutant clones in normal colon remain poorly characterised, and it is unknown whether some clones already exhibit phenotypic features associated with a higher risk of transformation. Detecting the molecular signatures of preneoplastic clones may offer a route to stratify early clonal expansions by their likelihood of progression.

In this work we combine targeted amplicon sequencing, mathematical modelling and spatial transcriptomics to quantify the prevalence, behaviour and evolutionary fate of cancer driver mutant clones in the normal human colon. We extend the catalogue of detectable mutations to include variants in *APC*, *KRAS*, *TP53*, *FBXW7* and *CTNNB1*. Monoallelic *FBXW7* (R465C) and *KRAS* (G12) missense mutations are under positive selection through distinct clone dynamic biases, whereas monoallelic *APC* and *TP53* inactivation appears selectively neutral. Modelling predicts that selection alters evolutionary cancer trajectories favouring *APC*-first pathways in older patients while allowing multiple routes, including *KRAS*-first, in younger individuals. Spatial transcriptomics identifies a subset of KRAS-mutant clones co-expressing gastric and intestinal markers indicating altered differentiation states that may increase transformation potential. Together these data demonstrate multiple evolutionary paths to CRC arising in normal tissue and underscore the need to stratify early driver mutations by cancer risk.

## Results

### Strategy for detecting mutant clones in the normal colon

**Fig. 1:**
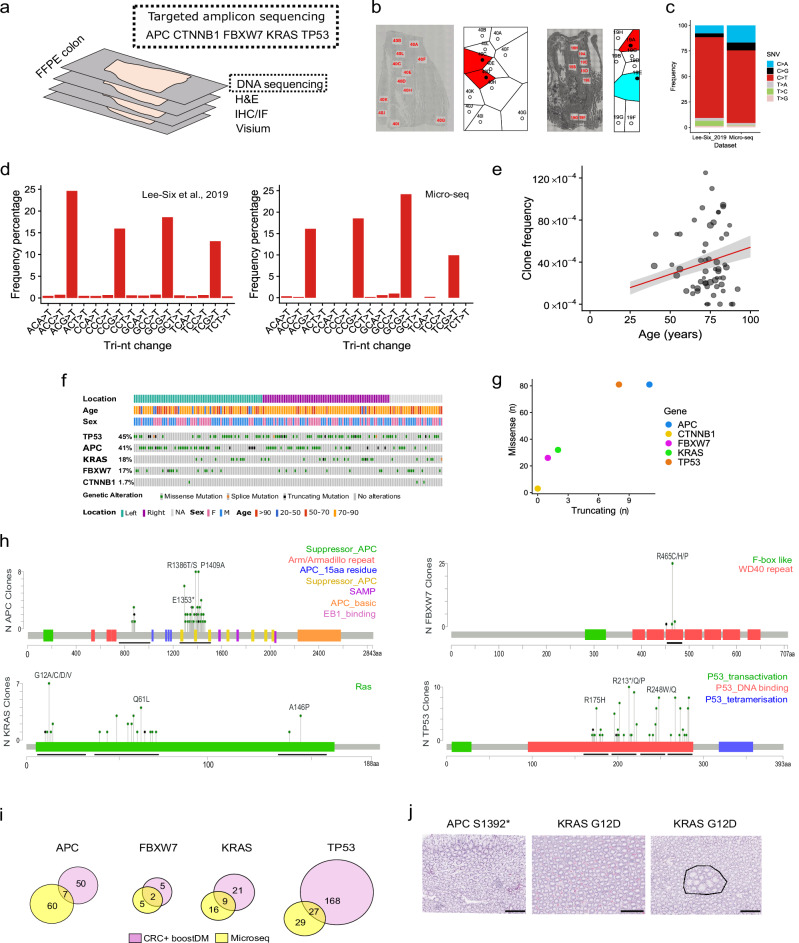
Presence of cancer-driver clones in normal human colon. **a** Experimental outline and use of serial sections. **b** Maps of laser-capture micro-dissected patches (Micro-seq) and respective tessellation plots generated using the Voronoi algorithm. Left: example of FBXW7 R465C mutation in adjacent patches counted as single clone (single colour). Right: example of TP53 G245S mutation in non-adjacent patches counted as two independent events (cyan and red). Scale bar indicates 750 μm. **c** Percentage frequency of type of single nucleotide variant (SNV) changes for Lee-Six et al.^1^ (*N* = 20,581 SNVs) and Micro-seq datasets (*N* = 289 SNVs corresponding to 285 clones). Normalised by trinucleotide abundance within the respective panel. **d** Individual trinucleotide changes for C > T mutations for Lee-Six et al.^1^ and Micro-seq datasets from (**c**). **e** Clone frequency for all clones detected in Micro-seq plotted against age. Slope: 5.12 × 10^−5^, y intercept = 3.27e-04. Each dot represents a patient (*N* = 56). Linear regression fit generated using RHClones R package. Solid line represents median and shading indicates the 2.5th and 97.5th quantiles of *N* = 1600 samples capturing the 95% credible interval of the posterior distribution. **f** Oncoprint for all detected mutations using Micro-seq with metadata. **g** Number of missense and truncating clones found per target gene. **h** Lolliplots for all detected SNVs in a gene plotted on protein domains. Top to bottom: *FBXW7*, *TP53*, *APC*, *KRAS*. Black lines under protein domains indicate amplicon coverage. **i** Venn diagrams of unique amino acid changes found in Micro-seq and CRC + boostDM (colorectal cancer data from COSMIC and all possible mutations assigned to have an oncogenic potential by boostDM described in Muiños et al.^23^). **j** Examples of detected cancer-driver clones in the 200-crypt sequenced areas shown in H&E serial section. Areas with *APC* 1392* and first *KRAS* G12D clones present no change in morphology. The dotted line in the second *KRAS* G12D area indicate abnormal *KRAS* mutant crypts. Representative images from *n* = 11 *APC* mutant clones and *n* = 18 *KRAS* mutant clones, 2 of which had abnormal morphology (one shown here on the right). Images acquired using ×2 magnification. Scale bar indicates 200 μm. Source data are provided as a Source data file.

In the colonic epithelium advantaged mutations can be positively selected due to two independent processes^4,13^. In the first, cells that acquire de novo mutations, along with their subsequent progeny (clones), compete with neighbouring wildtype crypt cells to become permanently fixed by completely populating individual glands in a process known as monoclonal conversion^14–16^. In the second, mutant clones advantaged in the process of gland replication or fission generate large multicrypt patches over time^17–20^. To assess the optimal conditions for detecting cancer driver events promoting either process we elected for a highly targeted, replicate-based sequencing analysis of the most frequently mutated regions of genes commonly found in CRC (hotspot regions in *APC*, *KRAS*, *TP53*, *FBXW7* and *CTNNB1*) that could be applied to FFPE tissue across three different scales, Micro-seq, AceStrip-seq and Section-seq, ensuring both high sensitivity of detection and wide tissue coverage (Supplementary Fig. 1 and 'Methods'). This is contrasted with whole genome sequencing of ~1000 individual crypts adopted by Lee-Six et al., the high genomic resolution of which can call more mutations per crypt while likely missing rare, founder events of expanded mutant clones. The use of serial sections adjacent to those used for DNA-sequencing allowed histological assessment and spatial mapping of clones in situ for downstream analysis (Fig. 1a).

### Micro-seq is optimal for mutation detection

Analysis of Micro-seq data from 384 samples (56 patients, 40–92 years old) each containing around 200 laser-capture micro-dissected crypts (*N* = 76,800 crypts in total) identified 289 single nucleotide variants (SNVs). AceStrip-seq (43 patients, 754,188 crypts) and Section-seq (189 patients, 4,114,532 crypts) identified 571 and 76 SNVs, respectively. Comparison of the number of SNVs detected per crypt for each method reveals that Micro-seq (3.7 × 10^−2^ SNVs/crypt) is the most efficient by several orders of magnitude (AceStrip-seq and Section-seq detected 1 in 7.5 × 10^−4^ and 1.9 × 10^−5^, respectively).

Section-seq involves whole area FFPE sections that have large but variable numbers of transected crypts which we have previously used to detect large expansion of *KRAS* mutant clones. The low frequency of SNVs detected across the amplicon panel by Section-seq suggests that such large expansions are relatively rare events. The lower frequency of SNV calls for AceStrip-seq compared to Micro-seq indicates that despite using samples systematically excised from whole sections to contain around 1000 crypts per sample, many events are still falling below the threshold for detection. Consequently Micro-seq data was selected as the optimal modality for SNV detection.

### SNVs accumulate with age in the normal colon

The 384 Micro-seq samples, each containing around 200 crypts, were laser captured from FFPE sections and included multiple samples from each patient. Spatial mapping of the 289 Micro-seq mutation calls common to subsamples of FFPE sections were conservatively assumed to be a single clonal event if derived from neighbouring samples as defined by computing a Voronoi tessellation for each section (Fig. 1b, 'Methods' and Supplementary Table 1), identifying a total of 285 putative mutant clones. Mutations across the amplicon panel exhibited a signature similar to that derived from coding mutations found previously by whole genome sequencing of individual crypts^1^ and dominated by CpG > T changes characteristic of the clock-like SBS1 signature^21,22^ (Fig. 1c, d and Supplementary Fig. 2a, b). Although known to include artefacts associated with formalin fixation the dominance of SBS1 is an expected feature of the mutational signature of the renewing epithelia, including the colon^1^. Further, despite the small size of our amplicon panel (1180 bp), an increase in SNVs with age was observed (5.11 × 10^−5^ per crypt per year; 95% credible interval (CI) = 3.8 × 10^−5^–6.89 × 10^−5^) (Fig. 1e), providing strong evidence that most mutations are of true biological origin. This equates to 2.16 exome mutations per crypt per year (95% CI = 1.63–2.91) which is comparable but slightly higher than that derived from the Lee-Six et al. data (Supplementary Fig. 2c, d). Also consistent with previous observations^1^, Micro-seq revealed a 20% higher rate of SNV age-accumulation on the right side of the colon compared to the left (Supplementary Fig. 2e).

### Cancer driver mutations are found in histologically normal tissue

Mutations were observed in all interrogated genes (Fig. 1f, g). However, for *CTNNB1* only four mutations (including G34V, which is frequently observed in CRC) were detected and this gene was therefore excluded from further downstream analyses. Notable were recurrent missense mutations of amino acid positions R465 in *FBXW7* and G12 in *KRAS* (Fig. 1h). *TP53* mutations were recurrently detected at positions R175, R213 and R248, which are also hotspot regions in CRC. In *APC* recurring truncating mutations included E1353*, Q879* and S1392*, while some missense recurring mutations were also identified (P1409A, R1386T). A single R876* truncating mutation, one of the most common *APC* mutations in CRC, was also detected. We attribute the detection of these additional mutations compared to previous studies^1^, to our screening strategy, which includes sequencing of a near 80-fold higher number of crypts.

Mutations detected with Micro-seq were compared with those found in CRCs (COSMIC data) and predicted to have oncogenic potential by boostDM, a machine-learning model assessing SNVs in cancer genes^23^. For all five genes a subset of 45 CRC cancer driver mutations defined in this way were detected in normal tissue (Fig. 1i). These corresponded to 11 *APC*, 24 *FBXW7*, 18 *KRAS* and 190 *TP53* cancer-driver mutant clones. Importantly, the same and some additional (e.g. APC R1450*) cancer driver mutations were also detected using AceStrip-seq and Section-seq, but to lower frequencies (Supplementary Fig. 3).

Regions of tissue containing clones with cancer driver mutations from the Micro-seq were reviewed histologically. Most associated with histologically normal tissue (Fig. 1j). However, two of 18 *KRAS* mutant clones (one with a G12D and the other with a G12C mutation) associated with small epithelial foci with enlarged crypts, typical of hyperplastic polyps. Importantly, all the *APC* mutant clones with truncating mutations were found in histologically normal colonic epithelium, highlighting that *APC* mutant glands can also be found in individuals without any genetic predisposition and manifest with no associated morphological change.

### Evidence of positive selection for *KRAS* and *FBXW7* missense mutations

**Fig. 2:**
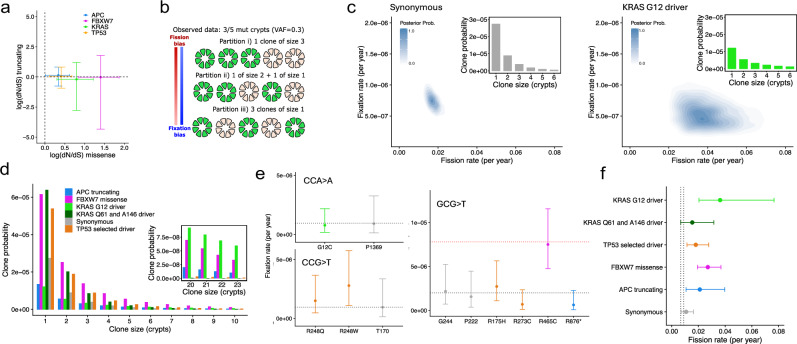
Evidence for positive selection and inference of clone dynamics. **a** dN/dS selection coefficients for missense and truncating mutations in *APC*, *KRAS*, *FBXW7* and *TP53* plotted on a log scale with upper and lower 95% confidence intervals (missense [centre, lower, upper], truncating [centre, lower CI, upper CI] = [0.331, 0.013, 0.618], [0.111, −0.780, 0.799]; [0.788, 0.295, 1.211], [−0.222, −2.81, 1.179]; [1.390, 0.839, 1.856], [−0.048, −4.352, 1.765]; [0.398, 0.099, 0.669], [−0.052, −1.127, 0.737]; respectively, *N* = 274 mutations). Dotted lines indicate an equal dN/dS ratio, indicative of no selection. **b** Cartoon illustration of possible partitions of three mutant crypts (equivalent to a VAF of 0.3 assuming five crypts in the sample) between mutant clones (green). In this case there are three different partitions of mutant clone sizes that equally well explain the observed data. If the mutant confers a bias in either fission or fixation this ascribes higher likelihood to the partitions with a larger clone size or larger numbers of clones, respectively. **c** Example density plots representing the posterior probabilities for fixation ($\Delta {C}_{{fix}}$) and fission ($\rho$) rates for synonymous and *KRAS* G12 driver mutations inferred from Micro-seq data. Insets show the corresponding clone size distributions where the probabilities of a mutant clone of a given size (x axis) is shown on the y axis. **d** Clone size distributions for each group of mutations used to infer clone dynamics from Micro-seq data: synonymous [all synonymous mutations]; *APC* truncating [S874*, R876*, Q879*, S1392*, E1353*, E1374*, C1410*]; *FBXW7* missense [H470D, R465C, R465P, R465H]; *TP53* selected driver [R273H, R248W, R175H, R248Q, R273Q]; *KRAS* Q61 and A146 [Q61L, A146P]; and *KRAS* G12 driver [G12D, G12C, G12V]. The Inset shows that mutations conferring higher fission rates drive expansion to larger clone sizes even though the probability of a clone is lower overall. **e** Individual fixation rates for mutations within the same trinucleotide context and therefore assumed to have the same background rate of spontaneous mutation. Black dotted lines represent averaged $\Delta {C}_{{fix}}$ values associated with synonymous (assumed neutral) probabilities of stem cell replacement, ${P}_{R}=0.5$, and bias in stem cell replacement conferred by the *FBWX7* R465C mutation, ${P}_{R}=0.7$, in red dotted line. Data are represented as median values in the centre with error bars for 2.5th and 97.5th quantiles of *N* = 2000 samples capturing the 95% credible interval of the posterior distribution. **f** Fission rate estimates for all groups of mutations inferred assuming individual fixation rates for each unique mutation compared with black dotted lines indicating estimates for homoeostatic rates of clonal expansion reported previously (Baker et al.^20^ and Nicholson et al.^4^). Data are represented as median values in centre with error bars for 2.5th and 97.5th quantiles of *N* = 2000 samples capturing the 95% credible interval of the posterior distribution. Source data are provided as a Source data file.

To formally assess whether mutated genes are under positive selection the ratio of non-synonymous to synonymous mutations was analysed using the dN/dS method adapted to the region of the genome targeted in Micro-seq^24^ ('Methods'). During dN/dS mutations are grouped to estimate background mutation rates for each trinucleotide context, which also controls for artefacts within a particular trinucleotide signature. This revealed that missense mutations in *FBXW7* and *KRAS* are under strong positive selection (selection coefficients >2 and adjusted *q* values, 3.2 × 10^−8^ and 1.8 × 10^−4^, respectively). No genes showed evidence of positive selection for truncating mutations (Fig. 2a). Among the most frequent missense mutations are G12D/C/V, Q61L and A146P in *KRAS* and R465C in *FBXW7*, suggesting these amino acid changes are the main drivers of positive selection for mutations in these genes.

### *FBXW7* and *KRAS* mutations confer biases to clone dynamics

We next sought the nature of the biases in the dynamics of cancer driver clones. Increased tissue representation of colonic clones is achieved by elevated rates of monoclonal conversion (fixation rate, $\Delta {C}_{{fix}}$) or crypt fission ($\rho$). To infer the effective values of $\Delta {C}_{{fix}}$ and $\rho$ associated with groups of mutations variant allele frequencies (VAFs) were used to estimate the number of mutated crypts contained within each Micro-seq sample and combined into a statistical model that considers all possible mutant clone sizes consistent with the observed data (Fig. 2b and 'Methods'). For example, compared to grouped synonymous mutations (20 unique mutations), where observation of many more events contributes to a higher value of $\Delta {C}_{{fix}}$, the group of *KRAS* G12 driver mutations (three unique mutations: G12D, G12C and G12V) presented with a much higher inferred value of $\rho$, reflecting a bias in crypt fission that drives clonal expansion and results in heavier tails in the predicted clone size distribution, corresponding to larger mutant clones on average (Fig. 2c, d).

Inferred $\Delta {C}_{{fix}}$ values depend on both the de novo mutation rate of the trinucleotide contexts in which they occur and the conferred probabilities of mutant stem cell replacement (fixation bias). Thus, mutations within each group were assessed for their fixation biases individually by inferring individual $\Delta {C}_{{fix}}$ values and comparing these across mutations that arise in the same trinucleotide context, assuming they share the same de novo mutation rate. *FBXW7* R465C was the only mutation exhibiting a substantially higher individual $\Delta {C}_{{fix}}$ value than other mutations within the same trinucleotide context (GCG > T) consistent with a higher probability of stem cell replacement or fixation bias (${P}_{R}=0.7$ for *FBXW7* R465C versus ${P}_{R}=0.5$ for a neutral mutation) (Fig. 2e).

Assigning individual $\Delta {C}_{{fix}}$ values further refined fission rate estimates for each mutation group confirming that elevated rates of clonal expansion are associated with *KRAS* G12 driver mutations as reported previously [3.9% of crypts undergoing fission per year (95% CI = 2.0–7.7%) compared to synonymous mutations (1.1%, 95% CI = 0.7–1.6%) and Q61L and A146P *KRAS* driver mutations combined (1.6%, 95% CI = 0.68–3.1%)]. *FBXW7* missense mutations were estimated to confer an intermediate increase in fission rate (2.5%, 95% CI = 1.9–3.7%), while credible intervals of fission rates for *APC* truncating and selected *TP53* driver mutations overlapped with those of synonymous mutations (Fig. 2f).

Taken together these results suggest that positive selection for *FBXW7* and *KRAS* missense driver mutations is conferred by two different modalities of altered clone dynamics: increased probability of stem cell replacement and fixation for *FBXW7* R465C, and highly elevated rates of fission for *KRAS* G12 mutations.

### Selection biases impact mutational burden

**Fig. 3:**
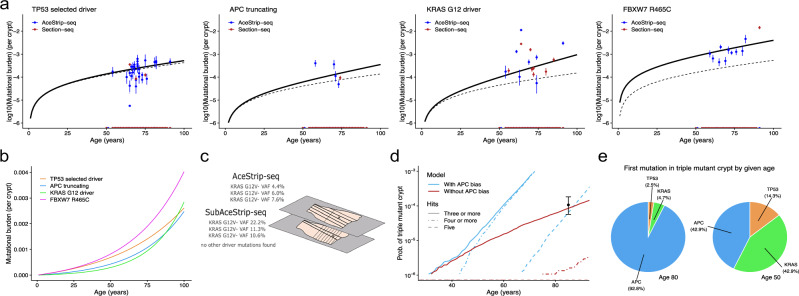
Relating mutational burden in normal tissue to the number of hits and order of events required for transformation. **a** Simulated mutational burden curves based on clone dynamics ($\triangle {C}_{{fix}}$ and $\rho$ values, bold line) and neutral dynamics (dashed line) inferred from Micro-seq data overlayed with observed mutational burdens of individual variants within each group from AceStrip-seq and Section-seq. Centres represent observed mutational burden at given age with error bars providing 95% Clopper–Pearson confidence intervals based on the total number of crypts sequenced from a total of *N* = 700 and 265 AceStrip-seq and Section-seq samples, respectively. Numerical values for the numbers of total crypts, numbers of mutated crypts, mutational burden and confidence intervals for each mutation at each given age are reported in the associated Source data file. Groups of *TP53* selected driver [R273H, R248W, R175H, R248Q, R273Q]; *APC* truncating [S874*, R876*, Q879*, S1392*, E1353*, E1374*, C1410*]; and *KRAS* G12 mutations [G12D, G12C, G12V]; FBXW7 missense [R465C]. **b** Total mutational burden curves on one scale for comparison between groups. **c** Cartoon schematic of the strategy for identifying co-occurring driver mutations in subAceStrip-seq applied to serial sections mapping to AceStrip-seq samples where large fields of mutant clones were detected. No putative driver mutations were found to co-occur within the fields of KRAS G12V mutant crypts analysed. **d** Comparison of simulation models adapted from ref. ^25^ with and without a selective bias attributed to biallelic *APC* inactivation where the probability of a crypt harbouring a mutation in each of the three genes *APC*, *TP53* and *KRAS* (among those detected) and given number of total hits is predicted as a function of age. The lifetime frequency of a cancer with the same triplet of mutations is represented as a black point at age 85 with 95% Clopper–Pearson confidence intervals (centre, upper, lower = 1.07e-04, 1.83e-04, 5.70e-05; using data from *N* = 6075 cancers). **e** Proportions of first driver mutation (founder) event in triple mutant crypts predicted among individuals at age 80 (left) versus age 50 (right). Source data are provided as a Source data file.

To study the consequences of selection in the normal colon age-related mutational burden accumulation curves were predicted based on the clone fixation and fission rates inferred from Micro-seq data ('Methods') (Fig. 3). In parallel, as additional validation, the independent but more restricted data derived by AceStrip-seq and Section-seq was leveraged and compared to the Micro-seq derived predictions (Fig. 3a). For *KRAS* and *FBXW7* simulations including biases in fission (*KRAS* G12) and fixation (*FBXW7* R465C) performed considerably better at describing the data than those without selection (ratio of root mean squared error (RRMSE) with/without selection 0.68 and 0.42 for *KRAS* G12 and *FBXW7* R465C, respectively). This pattern was not observed with the *TP53* missense driver mutations and to a lesser extent with APC truncating mutations (RRMSE 1.03 and 0.82, respectively) (Fig. 3a). Therefore the predictions made were consistent across the three sequencing scales.

We then compared mutational burden curves between each group of detected variants, which revealed striking differences in mutational burden and expected prevalence of different driver events (Fig. 3b). For example, attributed to a higher fixation rate, 0.1% of all crypts are estimated to contain the *FBXW7* R465C driver mutation by middle age (age 55) compared to only 0.038% with any one of the *KRAS* G12 driver mutations (G12D, G12C, G12V), despite both events being positively selected. However, even though there are far fewer mutational events overall, the high fission bias associated with *KRAS* G12 mutations means their mutational burden will eventually surpass that of detected *APC* truncating or selected *TP53* missense driver mutations over the course of a lifetime (age 95). Selection biases therefore dictate the nature and availability of mutant crypts for neoplastic transformation as a nonlinear function of age.

### Driver mutation co-occurrence is incredibly rare in the normal colon

Given the high mutational burden of individual driver mutations predicted in normal colon we next considered mutational co-occurrence, since multiple driver events within the same crypt are necessary for cancer initiation^5^. Five of the 384 Micro-seq samples shared two instances of the driver mutations studied here but, in each case, the VAFs of both mutations were below 1%, indicating that they are small clones within the harvested 200 crypt LCM-area and therefore unlikely to have a shared clonal origin (*p* < 0.0001 assuming two or fewer crypts harbour either mutation) (Supplementary Table 2). To explore driver co-occurrence systematically across wider portions of tissue we therefore opted for further targeted sequencing and mutational calling by sub-slicing serial sections adjacent to AceStrip-seq samples where a driver mutation was detected (Fig. 3c and 'Methods'). Rationally, this approach would enrich for fields of tissue containing exclusively mutated crypts, making other driver mutations detected in the original samples attributable to co-occurrent events with a high degree of certainty. Three large fields of *KRAS* G12V (VAFs 22.16, 10.625 and 11.285%) driver mutations were identified and analysed this way, including one from a 40-year-old patient with exceptionally high mutational burden overall. However, no other driver mutation, which would suggest a possible sub-clonal origin, was identified within these fields (Fig. 3c). Co-occurrence of two or more of driver mutations from the same crypt thus appears to be an incredibly rare event in normal tissue, even on the background of exceptionally large mutant KRAS clonal expansions.

### Positive selection raises the predicted event threshold for CRC initiation

The number of mutational events required for CRC initiation has been predicted to be between three and five^6–9^. Since co-occurrence is rare in normal tissue, to place observed mutant clone dynamics in this context, a simulation-based approach was adopted using an established mathematical model for CRC initiation involving *APC*, *KRAS* and *TP53* driver mutations^25^. The model considers all possible routes through mutation space that generate a crypt with biallelic inactivation of tumour suppressor genes (*APC* and *TP53*) and monoallelic activation of the oncogene (*KRAS*) and relates the frequency of such crypts to the observed lifetime risk of CRCs containing the same collection of events. Here, the model was reparametrised using our inferred fixation and fission rates for groups of monoallelic driver mutations detected in *APC*, *TP53* and *KRAS* ('Methods'). Notably, there is uncertainty in the true selection bias of *APC* null clones, precluding an exact estimate for the number of hits required for CRC initiation. Therefore, for the remaining undetermined parameters associated with biallelic inactivation of *APC*, we considered two possible opposing scenarios: either no additional selection bias is conferred by a second hit in *APC* or both fixation and fission biases are highly elevated for *APC* null clones, as assumed in the original ‘classical’ model (extrapolated from data from adenomas in ref. ^25^). The simulations demonstrate that without a selection bias for *APC* null clones the frequency of triple mutant crypts (i.e. those carrying at least one detectible driver mutation in each of the three genes *APC*, *TP53* and *KRAS*) approximates to the frequency of CRCs containing the same mutations at age 85 (Fig. 3d), meaning that three or more hits would be sufficient to explain CRC incidence. In contrast, incorporating a selection bias for *APC* null clones results in frequencies of triple mutant crypts that exceeds the frequency of CRCs by several orders of magnitude, indicating that additional events—likely around five in total—are required for CRC initiation (Fig. 3d).

### Founder events vary with patient age

Next the simulations were used to explore the temporal ordering of mutations giving rise to triple mutant crypts (crypts containing at least one mutation in each gene *APC*, *TP53* and *KRAS*), as potential precursors to CRC. Based on previous mutational burden calculations (Fig. 3a) and attributable to a large fission bias, *KRAS* G12 variants drive the largest expansions of all clones containing a single monoallelic driver mutation. However, crypts containing three driver mutations co-occurring in all three genes are more likely to have first acquired two independent hits in *APC* by age 80, with 92% of such triple mutated clones arising with *APC* as the first, founder mutation (Fig. 3e). As previously described^25^ this preference in temporal ordering is attributable to higher selection bias associated with *APC* null clones arising early and which confers a greater chance to subsequently accumulate multiple driver mutations over the course of a lifetime. A relevant corollary given the increasing incidence of CRCs in younger individuals, is that at younger age clones with triple gene mutations are more likely to have a preferentially arisen from crypts where a *KRAS* G12 driver mutations occurred first (42.9% versus 4.7% of clones at ages 50 versus 80, respectively) (Fig. 3e). Together these simulations suggest that positive selection for loss of *APC* progressively favours an ‘*APC* first’ aetiology for CRC with age but that, in younger individuals, multiple routes are possible with *KRAS* and *APC* having roughly equal probability of being the founder driver mutation event in triplet mutant crypts at age 50.

### Spatial transcriptomics identifies *KRAS* mutant clones in situ

**Fig. 4:**
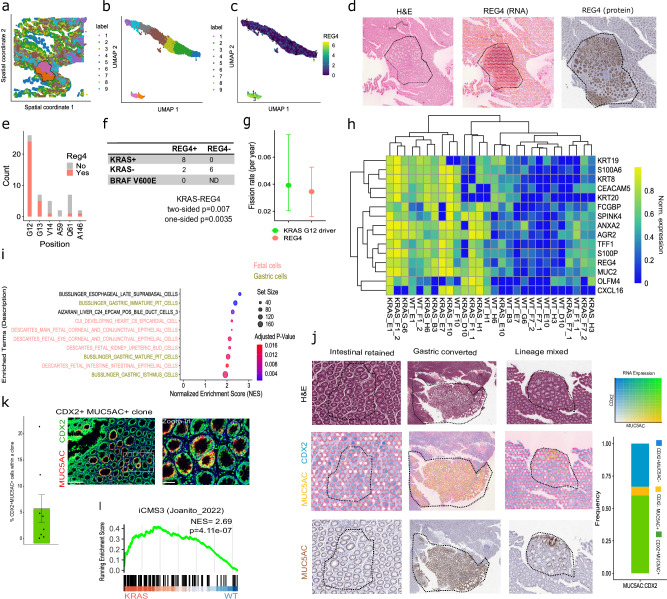
Spatial transcriptomics profiling of *KRAS* mutant clones. **a** Clusters overlaid on the spatial coordinates of a whole tissue section with the *KRAS* mutant clone annotated. **b** UMAP of the same section with annotated clusters. **c** UMAP of the same section with REG4 expression. **d** Enlarged regions containing the *KRAS* mutant clone depicted in (**a**) with H&E and REG4 RNA expression on RNA and IHC detection. **e** Type of mutations in *KRAS* amino acid positions associating with REG4 protein expression. Identified first using *KRAS* sequencing and then assessed for REG4 expression. **f** Contingency table for the 16 tissue samples profiled with REG4 IHC and paired sequencing for *KRAS* driver mutations reveals a strong statistical association between mutational status and expression profile (Fisher’s exact test). Conversely no *BRAF* V600E mutations were found in the REG4-positive regions. ND not done. **g** Fission rate inferred using REG4 immunohistochemistry (*N* = 90 clones and 53 patients) compared to *KRAS* G12 fission estimate from sequencing data. Data are represented as median values in the centre with error bars for 2.5th and 97.5th quantiles of *N* = 1600 and 2000 samples for REG4 and *KRAS* G12, respectively, capturing the 95% credible interval of the posterior distribution. **h** Heatmap with top 15 differentially expressed genes in *KRAS* mutant clones based on log fold-change. Shown as normalised expression per row. **i** Gene set enrichment analysis (GSEA) showing top 10 cell types enriched within all *KRAS* mutant clones (*N* = 14 clones). *P*-value and normalised enrichment score (NES) derived from two-sided permutation-based significance testing adjusted using the Benjamini–Hochberg false discovery rate (FDR) procedure and run using clusterprofiler GSEA function in R. **j** Examples of intestinal retained gastric converted and lineage mixed clones based on *MUC5AC* and *CDX2* RNA expression. Co-expression of *MUC5AC* and *CDX2* is indicated in green. Bottom: IHC for MUC5AC. Right: quantification of clone status based on RNA expression of *MUC5AC* and *CDX2*. **k** Immunofluorescence for CDX2 and MUC5AC on lineage mixed clones obtained using Akoya Polaris slide-scanner with 20× objective, 0.50 µm/pixel resolution. Scale bar indicates 200 µm. Inlet showing zoomed in version of clone with double positive cells in yellow with scale bar indicating 50 µm. Bar plot with quantification of the percentage of double-positive cells within a clone. *n* = 8 biological replicates. The plot shows the mean and the error bars indicate standard deviation. **l** Enrichment plot for iCMS3 signature from published data^35^. *P*-value and normalised enrichment score (NES) derived from two-sided permutation-based significance testing adjusted using the Benjamini–Hochberg false discovery rate (FDR) procedure and run using clusterprofiler GSEA function in R. Source data are provided as a Source data file.

The insight that CRC might have alternative founding events rather than necessarily proceeding from a crypt where *APC* inactivation occurred first, motivated us to look in more detail at clones carrying different driver mutations in normal tissue. Clonal expansions driven by monoallelic mutations may be pathologically indistinguishable from normal tissue yet possess inherent differences at the molecular level that impact their potential for accumulating additional driver mutations and propensity for neoplastic progression. To investigate whether monoallelic cancer driver clones can be phenotypically profiled in histologically normal tissue spatial transcriptomics (Visium, 10X) was performed on sections that were serial to those that were subsampled and containing *KRAS* [G12V, G12D], *TP53* [R175H, R273C], *APC* [R876*, E1353*], and *FBXW7* [R465C] mutant clones (Supplementary Fig. 4). No discrete clustering of the transcriptome from samples containing *APC*, *TP53* or *FBXW7* mutant clones was observed, which could be explained because a relatively small clone size does not drive sufficient transcriptomic change from the bulk to allow detection. However, regions of tissues containing mutant *KRAS* G12 driver clones formed clusters distinct from the bulk of the section (Fig. 4a, b and Supplementary Fig. 5a). Thus, among all monoallelic SNVs detected here, only *KRAS* G12 driver mutations are associated with a molecular phenotype notably distinct from that of normal colonic tissue.

### REG4 overexpression serves as a surrogate marker for *KRAS* activating mutations

The distinct phenotypic cluster found in tissue sections harbouring *KRAS* G12 mutant clones showed high expression of Regenerating family member 4 (*REG4*), allowing specific visualisation of clones in situ that was confirmed by immunodetection of the REG4 protein (Fig. 4c, d and Supplementary Fig. 5b). REG4 immunohistochemistry (IHC) further revealed that 68% (35/51) of *KRAS* mutant clones previously identified via DNA-sequencing were positive at the protein level, a proportion increasing to 92% (22/24) when only considering mutations at amino acid position G12 (Fig. 4e and Supplementary Fig. 5c). To assess the significance of this association paired REG4 IHC and *KRAS* amplicon sequencing was performed on 16 tissue samples not previously analysed by Micro-seq, identifying ten regions positive for REG4 and six negative, with each being laser-capture micro-dissected and sequenced for *KRAS* mutations (Supplementary Fig. 5d). *KRAS* mutations at positions G12 and G13 [G12D, G12C, G12V, G13D] were called in eight of the ten (80%) REG4 positive regions while none were called in the six (0%) REG4 negative regions. Fisher’s exact test shows a highly significant association (two-sided *p* = 0.007, one-sided *p* = 0.0035 testing enrichment of *KRAS* mutations in REG4 positive regions) (Fig. 4f). The REG4 positive regions that did not contain a *KRAS* mutation were also wild type for *BRAF* V600E which is similarly known to underly serrated polyp neoplasia that contain cells with a comparable REG4 positive phenotype^26^, although other mutations such as those in *NRAS* cannot be ruled out.

Previously we have exploited clone sizes quantified by direct visualisation with IHC to infer mutant clone dynamics^4^. Using positive REG4 expression as a surrogate for *KRAS* activation in the normal colon allows mutant *KRAS* G12 clone dynamics inferred using VAF-derived clone sizes to be compared with those obtained by direct visualisation using IHC. Inferences based on REG4 positive clone sizes indicated an elevated fission rate of 3.4% per year (95% CI = 2.3–4.7%, based on *N* = 90 samples from 53 patients) (Fig. 4g) comparable to that inferred for *KRAS* G12 driver mutations using Micro-seq data (3.9% per year). Together, the correspondence in the quantitative assessment of fission biases arising from *KRAS* activating mutations both supports positive REG4 expression as a surrogate of *KRAS* driver mutation and, more generally, the robustness of the sequencing data employed here in identifying such selection biases.

### *KRAS* mutant clones co-express intestinal and gastric lineage markers

The modelling predictions that *KRAS* G12 driver mutations are candidate founder events for triple mutant crypts especially in younger individuals, motivated us to investigate the molecular features of *KRAS* mutant clones and identify if any elements reminiscent of CRC were detectable at this early stage. Using positive *REG4* expression to identify 14 *KRAS* mutant clones captured by Visium profiling, we identified a *KRAS* mutational signature comprising 628 genes. Among the top differentially expressed genes were *MUC2*, *TFF1*, *S100P* and *SPINK4* (Fig. 4h). Gene set enrichment analysis (GSEA) showed enrichment for non-intestinal epithelial cell types including oesophageal, gastric and foetal cell types (Fig. 4i). Transcriptomic signatures associated with cell states expressing foetal or metaplastic markers ^27–30^ were similarly enriched within the *KRAS* mutant clone signature (Supplementary Fig 5e). Notably, there was no enrichment for classical intestinal stem cell signatures^30^ and secretory lineages of the colon^31,32^.

Gastric metaplasia has been previously reported in serrated polyps with *BRAF* mutations defined by *MUC5AC* upregulation and loss of the intestinal marker *CDX2*^26^. *KRAS* mutant clones expressed gastric markers including *MUC5AC* (Supplementary Fig. 5f) but complete gastric lineage conversion (MUC5AC^+^/CDX2^−^) was only observed in one clone, with 60% of *KRAS* mutant clones co-expressing *MUC5AC* and *CDX2* (Fig. 4j). Immunofluorescence staining of eight MUC5AC/CDX2 co-expressing KRAS mutant clones highlighted that in all the clones around 5% *KRAS* mutant cells were simultaneously expressing gastric and intestinal markers (Fig. 4k). In the stomach and oesophagus comparable co-expression of tissue specific markers has been described as incomplete metaplasia or lineage confusion and has been associated with a high risk of progression to dysplasia and cancer^33^. Although the lineage mixing seen here could be a transient state, to our knowledge, the phenomenon has not previously been described in normal colonic epithelium.

Finally, we queried the relevance of this mutant *KRAS* signature for CRC. Gastric metaplasia has been linked to the iCMS3 CRC transcriptional subtype where *REG4* and *SPINK4* are defining signature genes of this classification, and among which there is a known enrichment of *KRAS*-mutated tumours^34^. Here, we observed enrichment for CMS3/iCMS3 markers in *KRAS* mutant clones (Fig. 4l), suggesting that these iCMS3 profiles may be a consequence of an early *KRAS* driver mutation event conferring gastric features retained throughout the evolution of this subset of CRCs.

## Discussion

Using a highly targeted replicate-based amplicon sequencing approach, the list of cancer driver mutations found in the normal colon is now extended to encompass key CRC mutations, including R876*/R1450* in *APC*, R465C in *FBXW7*, G12C/D/V in *KRAS* and R175H/R248Q/W in *TP53*. Notably truncating *APC* mutations were found in histologically normal tissue in individuals without genetic predisposition to familial CRC.

Monoallelic mutations in *KRAS* and *FBXW7* but not *APC* or *TP53*, were found to display strong positive selection in normal colonic epithelium mediated by different clonal behaviours. *FBXW7* R645C mutations are favoured by biased stem cell competition leading to increased rates of fixation within the crypt. Clones carrying *KRAS* activating mutations at amino acid position G12 preferentially expand via increased rates of crypt fission. Previously, in mice, *KRAS* mutations have been shown to have biases in both clone fixation and expansion^19,35,36^. Here, we did not find any clear evidence for the former in the human epithelium, suggesting a potential species difference in impact, but comparable data in humans remains limited. Given their selection bias in normal tissue both *KRAS* activating and *FBXW7* R465C mutations have increased bioavailability as early events in CRC, while the neutral outcomes associated with single hit *APC* truncations and mutations of *TP53* suggest their cumulative mutational burden is dictated primarily by their susceptibility to mutational processes active in the colonic epithelium. Therefore, *APC* requires biallelic inactivation to be positively selected, unlike other tumour suppressors with haploinsufficient roles in the colonic epithelium^37^. However, our inference of fixation biases was limited by the paucity of detected *APC* truncating mutations, so this interpretation would benefit from additional appropriate mutation data.

Previous estimates suggest that the transformation rate of a crypt carrying any single cancer driver mutation is very low—approximately one in three million—highlighting the difficulty of reconciling the mutational burden of individual driver events with cancer incidence^1^. For CRC evolutionary models and epidemiological data indicate that between three and five co-occurring driver mutations are required for initiation^6–9^. However, crypts carrying two or more canonical CRC driver mutations are exceedingly rare in normal colon, raising questions about how these combinations arise during early tumorigenesis. Historically, the near ubiquity of biallelic *APC* inactivation in CRC has positioned *APC* loss as the founder event in most tumours. In support of this, simulations of driver mutation co-occurrence and temporal ordering indicate that if selection for biallelic *APC* loss strongly outweighs the more modest biases associated with monoallelic *TP53* missense or *KRAS* G12 mutations, then *APC*-null crypts are far more likely to acquire additional drivers over a lifetime. However, our simulations also revealed that in younger individuals, where the window for clonal evolution is shorter, alternative evolutionary routes emerge, where a *KRAS* founder event is equally probable within triple mutant crypts, particularly relevant in the context of rising incidence rates of early-onset CRC^10^.

A major uncertainty in predicting the extent to which *APC*-first evolutionary pathways dominate CRC initiation is the true magnitude and timing of the selection bias conferred by biallelic *APC* loss. Current estimates rely heavily on mouse models and adenoma tissue and typically assume that biased behaviour emerges immediately following loss of the second *APC* allele^20,25,36^. Yet, mouse studies suggest that *APC*-null phenotypes are density-dependent, whereby a critical threshold of *APC*-mutant crypts is required to initiate adenoma formation, with low densities failing to progress and high densities promoting aggregation and tumour initiation^38^. These findings imply that selection for *APC*-null clones may be context-dependent rather than intrinsic. Supporting this, a recent whole-genome sequencing study of individual crypts from familial adenomatous polyposis patients showed that somatic inactivation of the second *APC* allele almost always coincides with aberrant crypt foci (ACF) or adenomatous polyps and is rarely found in morphologically normal crypts^39^. The consistent morphological detectability of *APC* biallelic loss suggests that ACF size distributions could be used to quantify *APC*-associated selection bias in the human colon. Such quantitative insights will be essential to determine whether *APC*-first is an obligate pathway for CRC or one of multiple evolutionary routes that might be age-dependent.

Beyond their genomic landscape there are further phenotypic and external factors that dictate selection constraints and neoplastic outcome of mutant clones. For example, the heterogeneity in the phenotypic outcome of *KRAS* activating mutations documented here suggests that there are stable and inherent differences in the epithelial responses to identical mutations, such as their lineage state, which may further promote their transformative potential. These findings mirror issues with early detection efforts that require stratification of lesions by their risk of transformation for which pathological assessment might not be sufficient. Hyperplastic polyps with *KRAS* mutations and clear morphological changes are often thought not to progress to CRC, while *KRAS* mutant clones with no morphological change as seen here, present a transcriptomic signature resembling a specific subtype of CRC tumours (CMS3/iCMS3). This might provide a rationale for testing stratification strategies based on mutant *KRAS* phenotypes which could be facilitated in a clinical setting using REG4 immunodetection.

## Methods

### Human tissue

Histologically normal colon tissue samples were obtained from cancer patients (cancer normal) from Addenbrooke’s Hospital Cambridge, Norwich University Hospital and St James University Hospital Leeds under full local research ethical committee approval (REC 15/WA/0131 approved by Wales REC7 Research Ethics Committee, 06/Q0108/307 and 08/H0304/85 both approved by East of England-Cambridge East Research Ethics Committee as well as 12/LO/1217 approved by London–Bloomsbury Research Ethics Committee, respectively) according to the Health Research Authority institution. Colectomy specimens were fixed in 10% neutral buffered formalin. From areas of tissue without macroscopically visible disease mucosal sheets were removed from the specimens and embedded *en face* in paraffin (FFPE) blocks.

In total the study contains data from 222 patients with an average age of 68 years (23–91 years old), 106 females and 115 males. The overall patient cohort was on purposely gender balanced with a wide range of ages included. Patient consent has been obtained for publishing indirect identifiers including gender and age.

### Micro-seq

Sections were mounted on special slides with PET membrane (Zeiss, 15511306) that had been irradiated with UV for 30 min prior to cutting. LCM slides were dewaxed using Leica Multistainer ST5020. Laser-capture microdissection (LCM) was performed in the Leica LMD7000 laser microdissection system. Areas of 200 crypts were selected and harvested on lids of 0.2 mm radius tubes and were resuspended in 30 μl of Proteinase K buffer from the Arcturus PicoPure DNA kit (ThermoFisher, KIT0103). Lysis was performed for 5 h at 65 °C followed by 10 min incubation at 95 °C. Post-LCM the slides were scanned, and the location of each patch annotated.

### AceStrip- and subAceStrip-seq

Sections were mounted on 240 μm acetate sheets that had been pre-coated with 0.01% poly-L-lysine (P8920-100ML) for 5 min with agitation followed by rinsing with distilled water and allowed to dry completely. Acetate sheets were UV irradiated for 30 min prior to cutting. Post-cutting they were stored at 4 °C in a customised stand.

Dewaxing was performed manually using a customised stand (Supplementay Fig. 6). Acetate sections were soaked in xylene for 10 min then a series of reducing strengths of Ethanol (100, 75 and 50%) for 10 min each and finally rinsed in distilled water and allowed to dry thoroughly. Sections on acetate were placed on a plastic platform with a metal stencil on top. The stencil was designed and 3D printed to contain guiding slots with centres separated by 2 mm. Sections were sliced into strips vertically which sometimes were divided in two based on the amount of epithelium in each strip, with the aim to have around 1000 crypts in each strip for AceStrip-seq. Images of the strips were taken to note their location and spatial relationship. For subAceStrip-seq serial sections of the AceStrip-seq were used, whereby strips that were found to contain a mutation of interest were sub-cut into smaller pieces, containing around 200 crypts each.

The QIAmp DNA FFPE Tissue Kit (QIAGEN, 56404) was used according to the manufacturer’s instructions for DNA extraction (eluted in 40 μl water).

### Section-seq

Whole-section DNA sequencing was performed using DNA scrolls from whole sections. The QIAmp DNA FFPE Tissue Kit (QIAGEN, 56404) was used according to the manufacturer’s instructions for DNA extraction (eluted in 20 μl water).

### Library preparation and next-generation sequencing

Two sets of primers to span a hotspot area of interest at different sequences were designed using Primer3 for target amplification (Supplementary Table 3). The CS1 and CS2 Illumina adaptors were added at the start of the forward or reverse primers, respectively. Primer multiplex groups are listed in Supplementary Table 4.

PCR reaction components are described in Supplementary Table 5. PCR cycling was performed at 95 °C for 2 min for one cycle followed by 36 cycles at 95 °C for 10 s, 60 °C for 10 s and 72 °C for 15 s. The final cycle was followed by a 5 min extension at 72 °C.

Samples were barcoded using the Fast Start High Fidelity PCR System (Roche, Basel, Switzerland) according to the supplier’s protocol. After pooling and purification by Clean & Concentrator Kit (Zymo Research) and size selection by PippinBlue (Sage Science), samples were sequenced using 150-base pair (bp) paired-end sequencing with 15% PhiX in-house on the Illumina NovaSeq (SP) platform.

### Mutation calling

Mutation calling was performed using RePlow^40^ using dual replicate amplicon coverage. In brief, RePlow exploits replicate library preparations to separate the contribution of background errors (at the stage of library preparation) from sequencing errors (at the stage of library sequencing) using a statistical model, which calculates the total log ratio of probabilities for variant candidates compared to background errors across all replicates simultaneously. In doing so, adjusted VAFs (which were used as input for clonal inference as described in the corresponding 'Methods' section) are constructed by subtracting the contribution from sequencing errors, while background error profiles for the error model are calculated independently for the six base pair substitution types (A > C, A > G, A > T, C > A, C > G, C > T) across the targeted regions. For the calls with a positive log ratio of probabilities as returned by RePlow, an additional stringent filtering step was applied whereby we only retained variants that also had a positive log ratio of probabilities in both replicates individually. This was particularly important for excluding false positive calls at low VAF values which corresponds to small numbers of mutant crypts on a large wildtype background. For subAceStrip-seq, GCC > T mutations accounted for 2/3 of all calls, standing out when performing the mutational signature analysis and being unique to that sequencing scale. All calls in this trinucleotide context were removed as they were considered artefactual (possibly because sections were left on acetate for a longer time).

### Tessellation algorithm for clone calling

To parsimoniously assign multiple calls of the same mutation made from the same patient to individual clones we calculated a Voronoi tessellation of tissue sections using spatial coordinates of sites selected for Micro-seq. In this way, each Voronoi tile captures the largest area of unsampled tissue assigned to a given Micro-seq site. Multiple calls of the same mutation mapping to adjacent Voronoi tiles were then initially merged into the same clone (via a depth-first search algorithm) whereas those spatially separated by surrounding empty (for that mutation) Voronoi tiles were considered distinct clonal events. Counts of merged clones generated in this way were used as input for dN/dS. By comparison, all possible partitions of mutant crypts between clones, consistent with observed VAF values for these merged calls, were considered during clone dynamics inference, as described below.

### Mutation signature

A human colonic exome mutation signature was derived by collating the 20,581 SNVs identified within the genomic coding region of individual normal human colonic crypts by Lee-Six et al. The trinucleotide contexts of the SNVs were transformed to the conventional Sanger format and counts summed. This process was repeated for the Micro-seq mutation calls. Then normalised the Micro-seq and Lee-Six et al. SNV counts were normalised by the trinucleotide target frequency within their respective sequencing panels. Normalisation gives a relative frequency of each trinucleotide context SNV occurring and allows direct comparison between our mutation signature and that derived from Lee-Six et al. by accounting for differences in tri-nucleotide target abundance between the relatively small sequencing panel used here (1180 bp) and the whole exome.

### Quantifying selection using dN/dS

The latest version of the maximum likelihood implementation of dN/dS as originally described in ref. ^24^ and available in the R package dNdScv (https://github.com/im3sanger/dndscv), was applied to identify genes under positive selection. A custom reference object was built from the assembly GRCh38/hg38 by sub-setting the trinucleotide context-dependent substitution consequence matrix to the set of possible mutations based on the 1180 bp region of the genome selected for targeted sequencing in this study. Mutation counts used for obtaining d*N*/d*S* values reported in main text are from merged clone calls using a Voronoi tessellation as described in the above 'Methods' section but we repeated the analysis with the more conservative assumption of just one unique mutation/clone per patient to obtain comparable results (d*N*/d*S* coefficients > 2 and adjusted *q* = 4.0 × 10^−7^ and 2.0 × 10^−4^ for *FBXW7* and *KRAS* missense mutations, respectively). *CTNNB1* mutations were also included when running dN/dS but are not plotted here (not positively selected).

### Calculating stroma:epithelium ratio

Crypt to stroma ratio was calculated using serial H&E sections and Halo (Indica Labs) for image analysis (Supplementary Fig. 6f, g). A crypt to stroma classifier (random forest) was used and a cell segmentation algorithm (Multiplex IHC) to calculate the number of cells in the epithelium and the stroma. A single stroma:epithelium ratio was calculated per section for the Micro-seq data and an individual stroma:epithelium ratio per strip was calculated for Strip-seq. For Section-seq we assumed a stroma:epithelium ratio of 1:1. This was used to calculate the inferred patch size based on the variant allele frequency VAF accordingly: [2 × VAF × (1+ stroma/epithelium) × crypt *N*].

### Inference of clone dynamics from sequencing data

Previously estimates for the fixation and fission rates conferred by specific *KRAS* mutations (at amino acid positions G12 and G13) were obtained using a statistical model for observed mutational burden based on targeted sequencing data^3,4^. In that original statistical model all mutated crypts detected in a sample (calculated from observed VAF values) are attributed to a single clone. However, in practice any combination of individual clones with a total number of mutant crypts equal to that observed can equivalently explain the data, a possibility that we account for in the updated statistical model outlined here.

From the clone size distribution^4^ the probability of a mutant clone with $n$ mutant crypts at age $t$ is

1 $${F}_{n}\left(t\right)=\Delta {C}_{{fix}}\frac{{\left(1-{e}^{-\rho t}\right)}^{n}}{\rho n}$$

where $\Delta {C}_{{fix}}$ is the rate of monoclonal conversion (fixation rate) of mutant crypts and $\rho$ is the fission rate associated with that mutation. The probability of a crypt not being mutated is

2 $${F}_{0}\left(t\right)=1-\,\Delta {C}_{{fix}}t$$

Given an observed number of mutant crypts $m$ in a sample with $N$ total clones the likelihood of a particular partition of $m=\,{n}_{1}+{n}_{2}+\ldots+\,{n}_{M}$ across $M$ mutant clones $i={{\mathrm{1,2}}},\ldots,M$ (with ${n}_{i}$ the number of mutant crypts in clone $i$) is then

3 $$\left(N-M,{k}_{1},\ldots,{k}_{m},0\right)\, \sim \,{{\mathrm{Multinomial}}}\left({{{\bf{q}}}}\right)$$

where ${k}_{j}$ is the number of clones in the partition with ${n}_{i}=j$ and ${{{\bf{q}}}}=({F}_{0}\left(t\right),\,{F}_{1}\left(t\right),\,\ldots,{F}_{m}\left(t\right),{q}_{*})$ with ${q}_{*}$ chosen to normalise the multinomial probabilities to sum to one. Note that the partitioning ensures ${k}_{m}=1$ and ${k}_{j}=0$ for all $j\ne m$ if there is just a single clone with $m$ mutant crypts and so the original assumption used in can be seen as a special case of this more general model.

To calculate the full likelihood of observing a mutational burden of $m$ mutant crypts one must sum up the likelihoods of all possible partitions of $m$. Enumerating integer partitions leads to a combinatorial explosion in increasing $m$ but summing over partitions during parameter inference is computationally feasible provided $m$ remains sufficiently small. In our Micro-seq data all but one (a *KRAS* G12C mutation found in $m=94$ crypts) observed numbers of mutated crypts were less than or equal to $m=15$, which was therefore used as a maximum clone size during inference of clone dynamics. As described for the original inference method in Nicholson et al. a critical number of mutant crypts was calculated for samples where no mutations were detected to accommodate the probability of the total number of mutant crypts falling below the detection limit of sequencing. The priors used here for $\Delta {C}_{{fix}}$ and $\rho$ are the same as in Nicholson et al.

Since the current statistical model accounts for but cannot resolve the ambiguity concerning the precise partitioning giving rise to the observed number of mutant crypts in each sample inferred values for $\Delta {C}_{{fix}}$ and $\rho$ should be interpreted as effective in that they are the most consistent with the observed data and their 95% credible intervals capture all possibilities. Simulations confirmed that the partition-based model generates sensible estimates for effective fission and fixation rates in extreme scenarios where true fission rates are comparably low and true fixation rates are very high, which the original model fails to do by assigning all mutant crypts to a single clone (Supplementary Fig. 7). When applying the model to real data from groups of variants assumed to share comparable fission rates we considered two different regimes: either the group was assigned a single shared value of $\Delta {C}_{{fix}}$ or each variant was assigned its own $\Delta {C}_{{fix}}$ independently, as described in the main text. ${F}_{n}\left(t\right)$ was used to generate figures with illustrative clone size distributions for different mutation groups using inferred $\Delta {C}_{{fix}}$ and $\rho$ values with an average patient age of 60.

### Mutational burden simulations

Inferred fixation and fission rates were used to calculate the estimated and plot mutational burden for a group of mutations in normal colon $B\left(t\right)$ at a given age ${t}$ as described in ref. ^37^ according to the formula

4 $$B\left(t\right)=\,{\sum}_{n=1}^{\infty }n{F}_{n}\left(t\right)$$

Here ${F}_{n}(t)$ is the distribution for the probability of a patch of mutated crypts of size $n$ at age $t$ as described in the previous 'Methods' section. The mutational burden of a triple co-occurrence was then predicted assuming independence of the three individual events by multiplying the corresponding mutational burden curves together. To express mutational burden in terms of the number of mutant crypts per individual we used $4.2\times {10}^{7}\cdot B\left(t\right)$, assuming a total number of 42 million crypts in the average human colon.

To simulate mutational burdens in the absence of selection we assigned the inferred fission rate for synonymous mutations to all groups of mutations and used the $\Delta {C}_{{fix}}$ estimate for synonymous mutations in the GCG > T trinucleotide context for *FBXW7* R465C. Experimental observations of mutational burden obtained from AceStrip-seq and Section-seq were calculated for each variant by dividing the estimated number of mutant crypts by the total number of crypts screened at a given age, excluding patients under the age of 50 to avoid anomalous data points associated exceptionally high mutational burden at a young age. Root mean squared errors were calculated based on the mean Euclidean distance between observed and predicted mutational burdens in the absence or presence of selection with the ratio of the latter to the former giving a measure of performance (ratio root mean squared error (RRMSE) = 1.0 if model with selection describes data equally well as model without, while RRMSE < 1.0 when the model with selection performs better).

### Simulations and comparisons with cancer incidence rates

The mathematical model from Paterson et al. was modified to incorporate clone fixation and fission rates inferred using the groups of *APC* truncating, *KRAS* G12 and selected *TP53* driver mutations detected in our study. Briefly, the original model was parameterised using gene-specific de novo mutation rates estimated based on the number of positions generating inactivating (for *APC* and *TP53*) and activating (for *KRAS*) mutations in the corresponding gene, combined with experimental data from a mixture of murine and human studies. Here, fixation and fission rates for monoallelic mutations in these genes were instead taken directly from our inferred clone dynamics, and the modified model should therefore be interpreted as one that further restricts the set of possible mutations to those detected via Micro-seq. Unmeasured parameters corresponding to the rate of loss of heterozygosity retained the same values used in the original model while the fixation and fission rates of clones with biallelic *APC* inactivation (and consequently crypts with biallelic APC inactivation and *KRAS* activation) were considered either: identical to those for monoallelic APC inactivation (without *APC* bias model); or unchanged from the original model (with *APC* bias model). Both models were simulated using default settings and optimised for threading across 120 HPC cores following the documentation on the code repository (https://github.com/chaypaterson/CRC_Initiation), first using the tau-leaping version of the stochastic simulation algorithm for calculating probabilities of mutant crypts by a given age and then in lineage mode for tracking ordering of different mutational events as described in the original publication^25^.

Probabilities of mutant crypts by a given age were compared with data from the following studies from cbioportal (https://www.cbioportal.org/)^41^ that had covered the regions included in the amplicon panel comprised the colorectal cancer dataset: Colon Cancer (Sidra-LUMC AC-ICAM, Nat Med 2023), colorectal adenocarcinoma (DFCI, Cell Reports 2016), colorectal adenocarcinoma (Genentech, Nature 2012), colorectal adenocarcinoma (TCGA, Firehose Legacy), colorectal cancer (CAS Shanghai, Cancer Cell 2020), colorectal cancer (MSK, Gastroenterology 2020), “colorectal cancer (MSK, JNCI 2021), metastatic colorectal cancer (MSK, Cancer Cell 2018), rectal cancer (MSK, Nature Medicine 2022), rectal cancer (MSK, Nature Medicine 2019). The frequency of a triple co-occurrence in CRC was calculated as the fraction of samples containing at least one mutation from each of the three corresponding sets of mutations, divided by the total number of samples (*N* = 6075) with 95% confidence intervals obtained using the Clopper–Pearson method. This frequency was then multiplied by the lifetime risk of CRC (5%) to estimate the lifetime incidence of CRC with that triple co-occurrence in the population.

### Immunohistochemistry/immunofluorescence

IHC was performed as previously described^4^. Briefly paraffin sections were deparaffinized and rehydrated using sequential xylene and graded alcohol washes, followed by heat-mediated antigen retrieval in 10 mM trisodium citrate buffer (pH 6.0). Sections were incubated in 3% hydrogen peroxide in methanol for 15 min and blocked with 10% donkey serum for 30 min. Sections were then incubated overnight at 4 °C with primary antibodies against REG4 (HPA, HPA046555, 1:1000, Polyclonal) and MUC5AC (Abcam, ab198294, 1:200, clone EPR16904). After washing slides were incubated for 40 min at room temperature with biotin-SP-conjugated AffiniPure donkey anti-rabbit secondary antibody (Jackson ImmunoResearch; 1:500 in PBS-T), followed by incubation with VECTASTAIN® Elite® ABC reagent (Vector Laboratories) for 40 min. Signal detection was performed using a liquid DAB+ chromogen system (Dako). Sections were counterstained with hematoxylin, dehydrated, cleared and mounted.

The IHC protocol was adapted for immunofluorescence without the hydrogen peroxide and the DAB steps and instead using secondary Alexa fluorophore conjugated antibodies (Thermo Fisher, 1:200) and DAPI (1 μg/mL). MUC5AC (Abcam, ab198294, 1:100, clone EPR16904) and CDX2 (Sigma, AMAB91828, 1:500, clone CL12974) primary antibodies were used. Slides were scanned on the Akoya Biosciences Vectra Polaris Automated Quantitative Pathology Imaging System (Perkin Elmer).

REG4 antibody has been validated by Human Protein Atlas. MUC5AC antibody has been validated by Abcam and has been cited in more than 10 publications. CDX2 antibody has been validated by Sigma using orthogonal RNAseq.

### Age accumulation and REG4 IHC-based inference

RHClones package (https://github.com/ElEd2/RHClones) was used to estimate the age-related slope of detected clones (*N* = 291). The same package was used to infer clone dynamics based on REG4 IHC data.

### LCM for REG4 positive samples

Serial sections of samples with identified REG4 positive regions were cut on LCM sections as previously described (Micro-seq section). REG4 positive regions together with some adjacent WT crypts to increase genomic material input, were cut and sequenced using next-generation sequencing for *KRAS* mutations (G12/G13, A146, Q61) using the previously mentioned primers (Supplementary Table 3). WT/REG4 negative samples were included for each patient to exclude any possible SNPs. For mutation calling a paired-end assembler for Illumina sequences, PANDAseq, was used to merge corresponding forward and reverse reads into an in silico amplicon^42^. The top ten different species that started and ended with the correct primer sequence were identified and their frequency was calculated. Mutated samples contained WT species together with mutant *KRAS* species.

### Visium slide processing and library preparation

Serial sections with identified mutant clones of interest from the MicroSeq and AceStripseq were cut with the ROI being placed at the centre of the glass slide. Slide processing and library preparation were performed according to the 10X Visium Cytassist FFPE protocol.

### Visium data analysis

Data alignment was performed using Spaceranger (10X). LoupeBrowser was used to draw around the mutant *KRAS* regions and matched WT and extract the barcodes. Spots with UM1 < 1000 genes <900 and mitochondrial gene percentage > 20% were filtered out from the analysis. Principal component analysis unsupervised clustering, differential gene expression analysis and dimensionality reduction was performed using the spatial experiment library in R.

The *KRAS* mutant signature was generated by compiling the top 400 differentially expressed genes from each of the 14 clones and selecting genes common to at least two clones (*N* = 628 unique genes). GSEA was performed using the cluster Profiler and msigdbr libraries in R.

Since we identified R465C to be associated with a strong selection bias we used the spatial localisation information of clones provided by Micro-seq and AceStrip-seq to perform dimensionality reduction analysis on the smaller regions containing FBXW7 R465C mutant clones. No distinct cluster appeared in such regions likely reflecting a lack of transcriptional alterations associated with the loss of one tumour suppressor allele and/or small clone sizes that precluded clustering.

### Statistical tests

All statistical tests were performed using default functions available in the R programming language. Fisher’s exact test was performed using the function fisher.exact() with default parameter values.

### Reporting summary

Further information on research design is available in the Nature Portfolio Reporting Summary linked to this article.

## Supplementary information

Supplementary Information

Reporting Summary

Transparent Peer Review File

## Source data

Source data

## Data Availability

The raw DNA amplicon-seq data generated in this study have been deposited in the NCBI database under accession code PRJNA1372027. The raw spatial transcriptomics data generated in this study have been deposited in the GEO database under accession code GSE312204. Processed data are available as supplementary data. Source data are provided with this paper.

## Code availability

All code for reproducing the analyses in this paper can be found at the GitLab repository https://gitlab.com/davidtourigny/crypt-clone-dynamics.
