# Supplementary material for: Clonal biases dictate availability of colonic cancer driver mutations for transformation
Source: Nat Commun. 2026 Apr 16;17:5295. doi: 10.1038/s41467-026-71944-5 (PMC13269798; doi:10.1038/s41467-026-71944-5)
Supplement: Supplementary file 1 — Supplementary Information [file 41467_2026_71944_MOESM1_ESM.pdf]

Supplementary Information

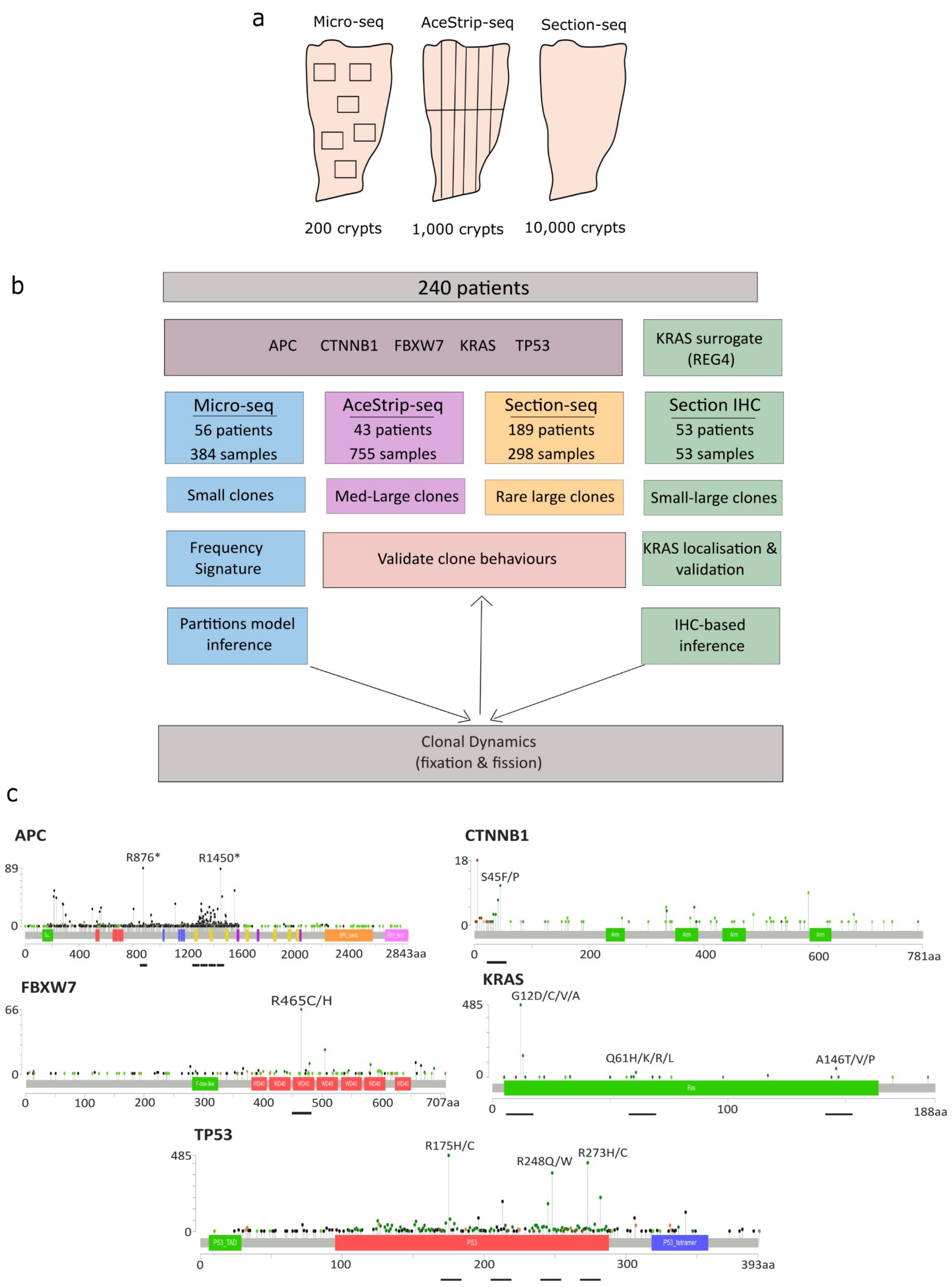

Supplementary Figure 1: Experimental set up. (a) Tissue processing of sampling scales. (b) Patient cohort used across different methodologies. From top to bottom: target genes, number of samples and patients, clone size target, purpose of method in the study, type of model used for inference and final output. (c) Lolliplot of colorectal cancer mutations across five sequencing target genes (cbioportal). Black lines under amino acid positions indicate amplicon panel coverage. Source data are provided as source data file.

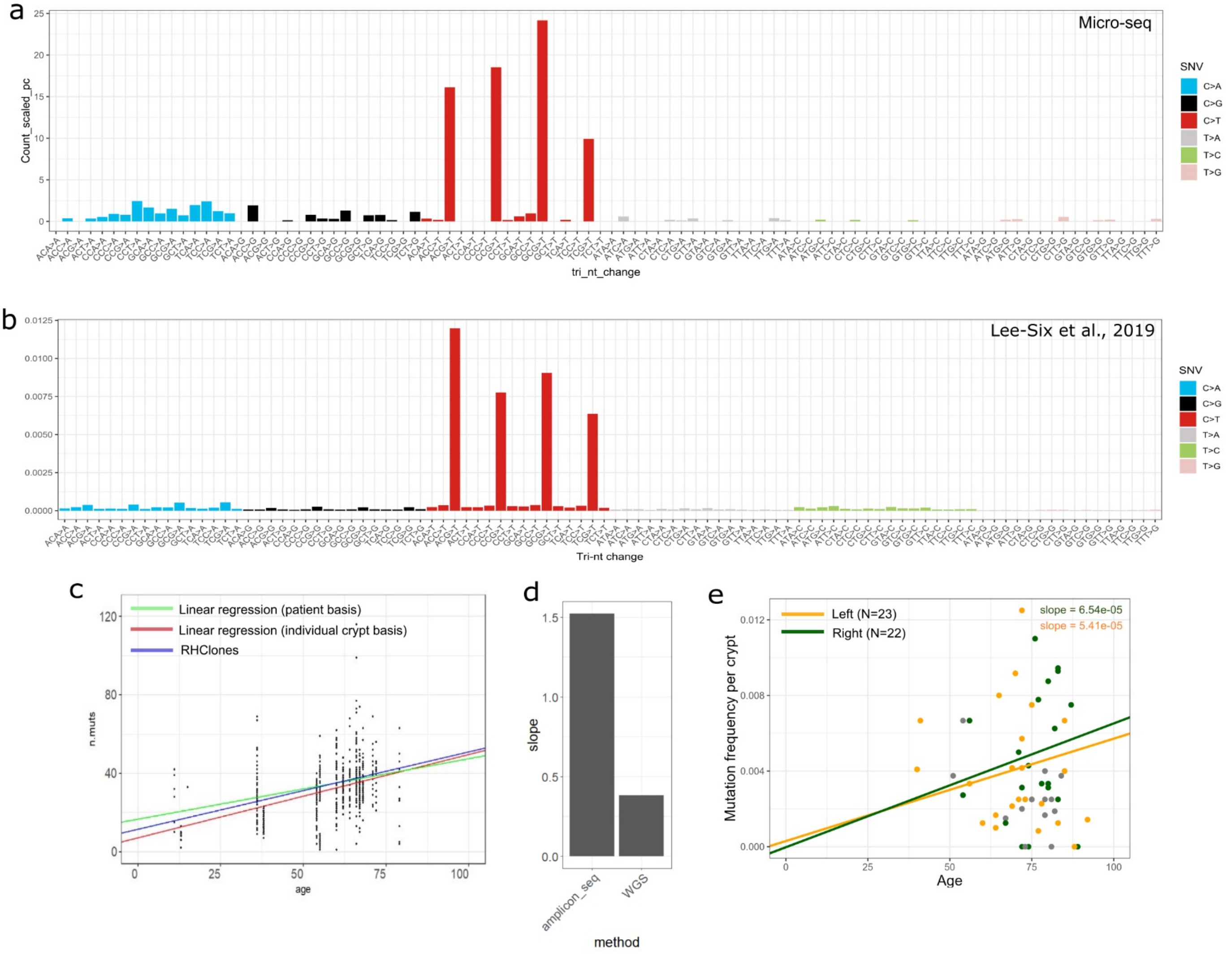

Supplementary Figure 2: Comparison of mutational signature and mutation accumulation with Lee-Six 2019 data. (a) Micro-seq data mutational signature. (b) Lee-Six data mutational signature. (c) Lee-Six slope based on whole genome sequencing data. Green line is inferred using standard linear regression (patient basis), red line is inferred using standard linear regression (individual crypt basis) and blue line is inffered using RHClones package. Red line: slope= 0.4527, y-intercept=7.016, Green line: slope= 0.3089, y intercept=16.51, Blue line: slope= 0.395, y intercept=11.376 (d) Lee-Six slope bsed on either their amplicon-seq or whole genome sequencing (WGS) data. (e) Comparison of Left vs Right side of the colon slopes from Micro-seq data. Source data are provided as source data file.

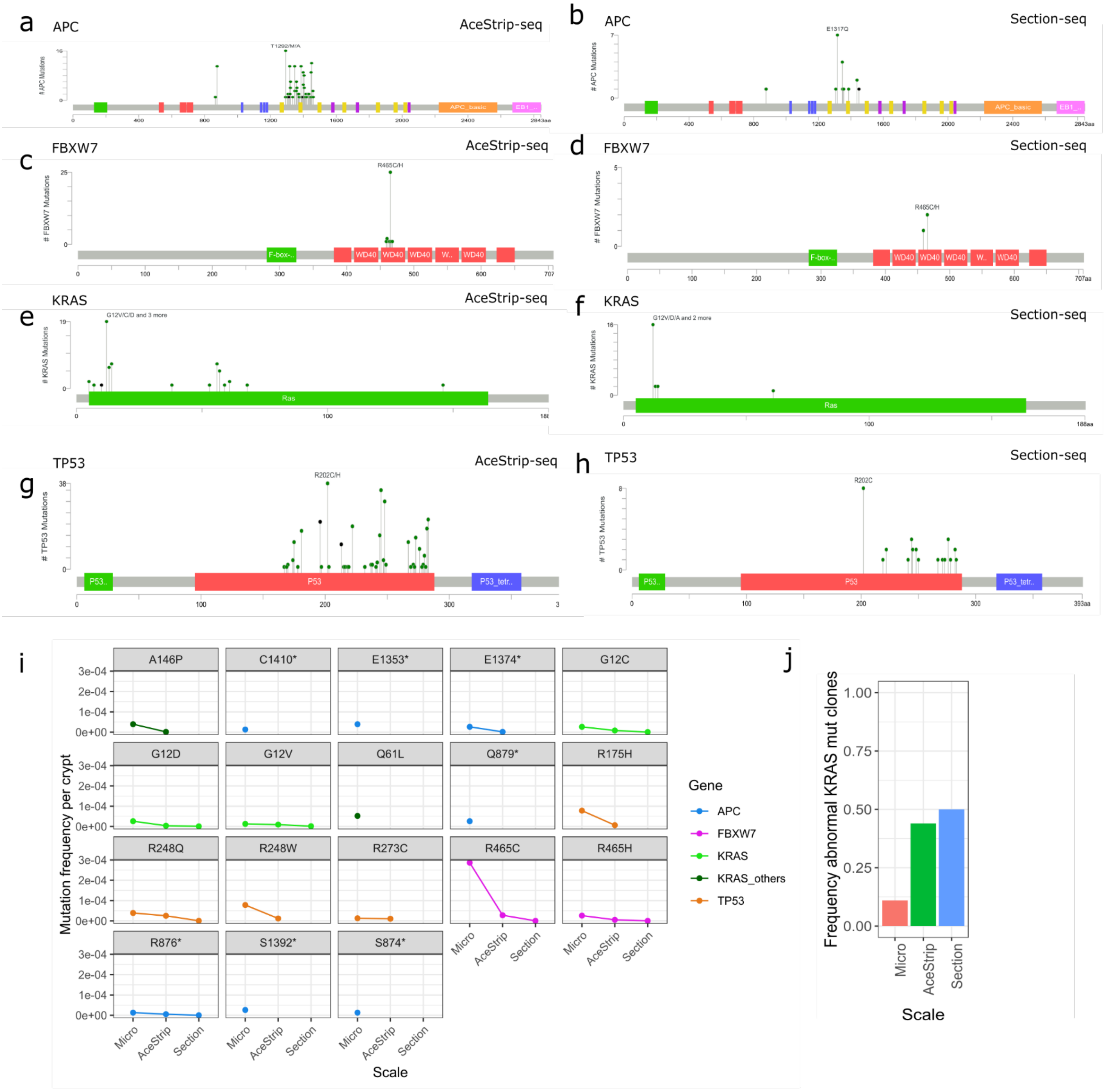

Supplementary Figure 3: AceStrip-seq and Section-seq data. (a-h) Lolliplots for detected mutations. (a-b) APC. (c-d) FBXW7. (e-f) KRAS. (g-h) TP53. (a,c,e,g) Ace-strip seq. (b, d, f, h) Section-seq. (i) Mutation frequency per crypt across scales for variants of interest first identified at Micro-seq. (j) Frequency of abnormal KRAS clones across scales (N=18 total Micro-seq, N=27 total AceStrip, N=16 total Sction-seq).
Source data are provided as source data file.

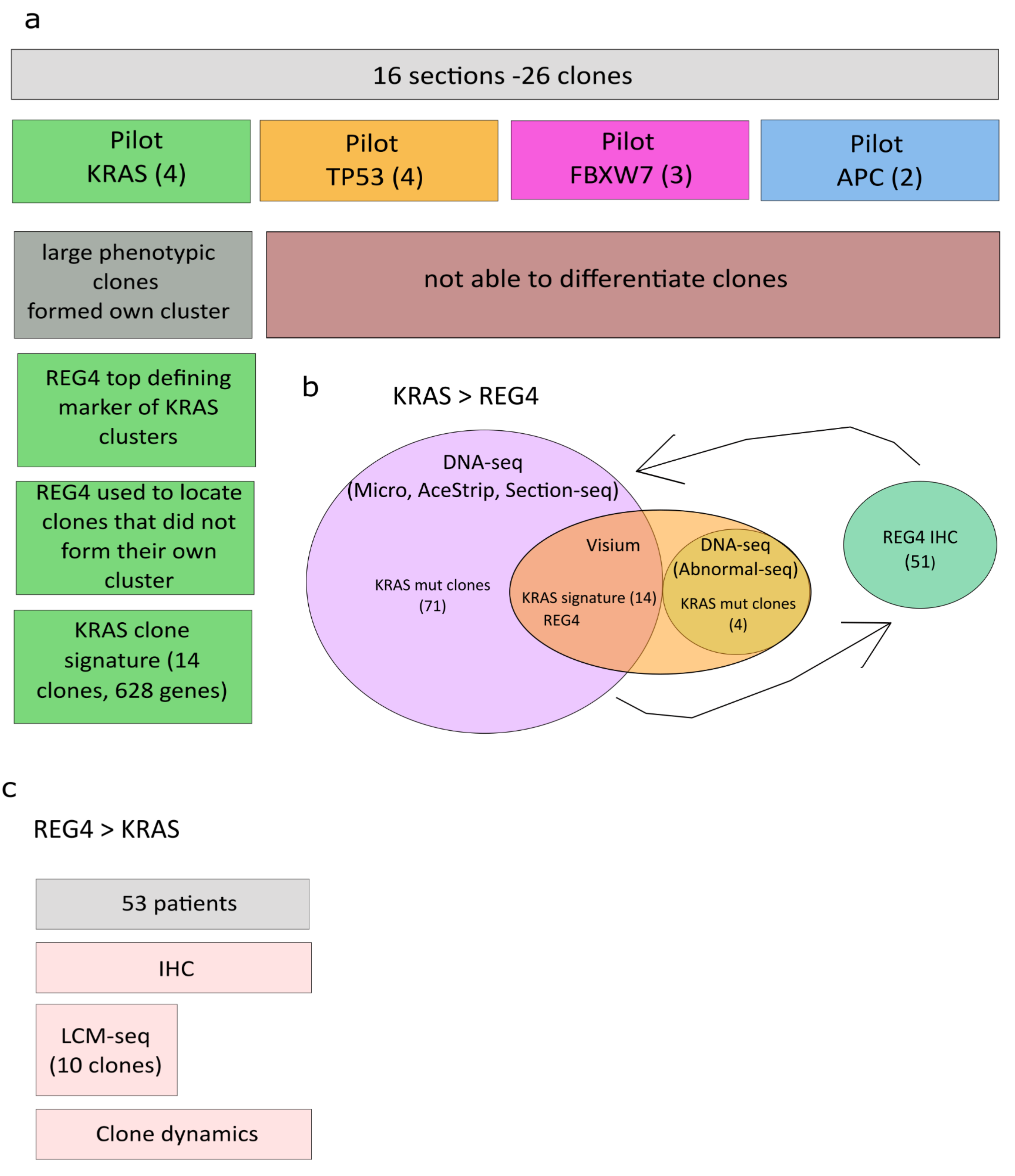

Supplementary Figure 4: Visium and KRAS-REG4 experimental set up. (a) Breakdown of Visium samples. Top to bottom: Pilot with number of clones in parentheses for each target gene. Only KRAS clones were followed up with more samples included after the pilot. Steps followed to generate KRAS transcriptomic signature. (b) identification of REG4 as a surrogate KRAS marker. KRAS mutant clones upregulate REG4. Number pf clones in parenthesis.(c) Validation that REG4 high clones in a different patient cohort that had not been previously screened for KRAS mutations. Laser-capture microdissection (LCM) and sequencing for KRAS G12, Q61 and A146P mutations was conducted. REG4 used to confirm clone dynamics inferred for KRAS mutant clones.

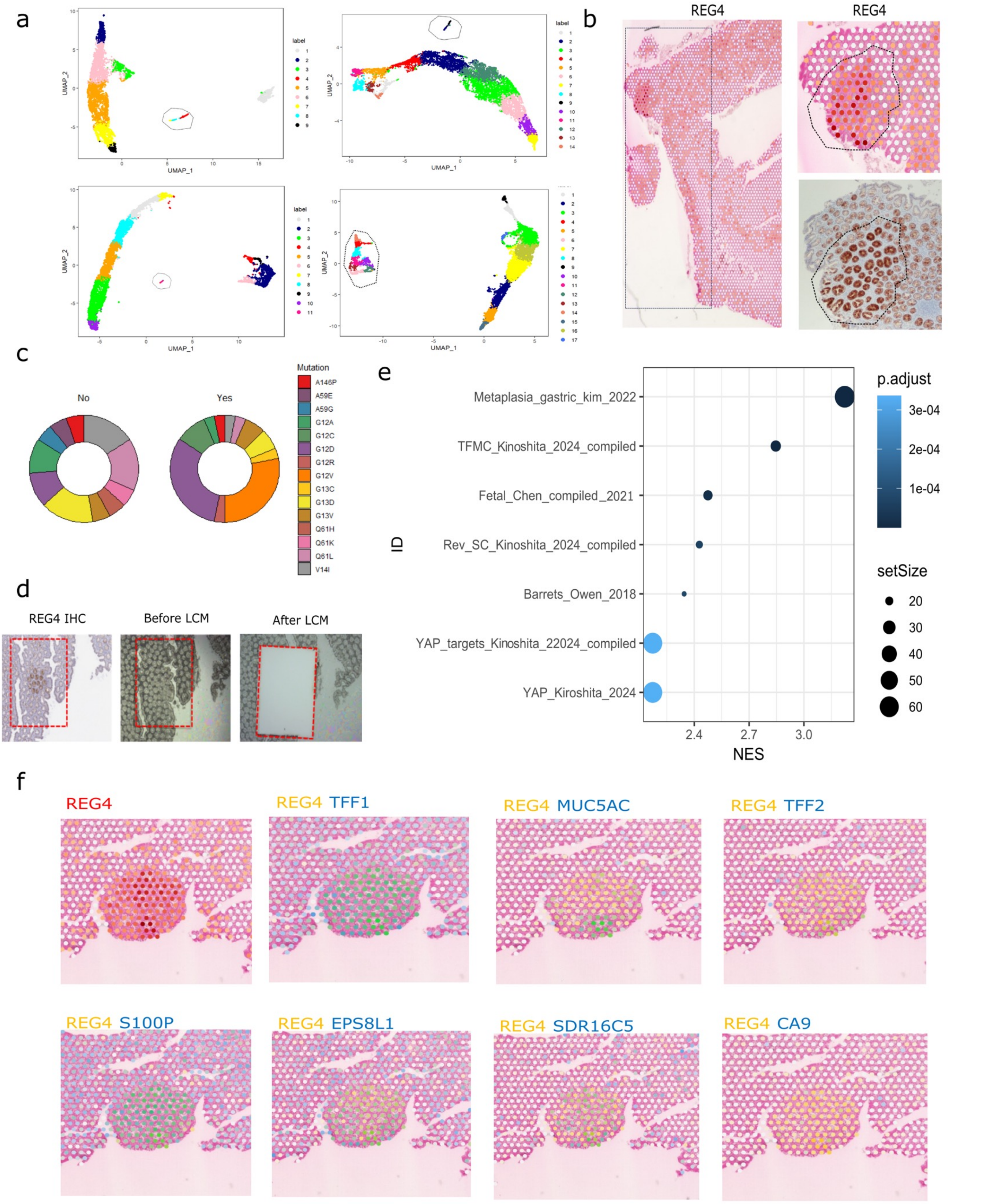

Supplemental Figure 5: KRAS mutant clone transcriptomic analysis and REG4 expression. (a) UMAP analyis of 4 independent sections with KRAS clones that form their own clusters. Annotation indicates KRAS clusters based on spatial location. (b) Left: Box indicates region expecting to have a KRAS mutation based on AceStrip-seq with REG4 RNA expression overlayed. Right: Zoom in REG4 positive region with RNA and protein expression top to bottom. (c) REG4 expression based on KRAS amino acid change. (d) Laser-capture approach of REG4 positive areas subsequently sequenced for KRAS (G12, Q61, A146 regions). (e) Gene Set Enrichment Analysis (GSEA) of KRAS mutant clones for previously published signatures relating to metaplasia and foetal genes (Owen et al., 2018, Kinoshita et al., 2024, Chen et al., 2021, Kim et al., 2022). (f) Expression of different gastric cell markers overlayed on REG4 expression that indicates KRAS mutant clone. Co-expression is seen in green.
Source data are provided as source data file.

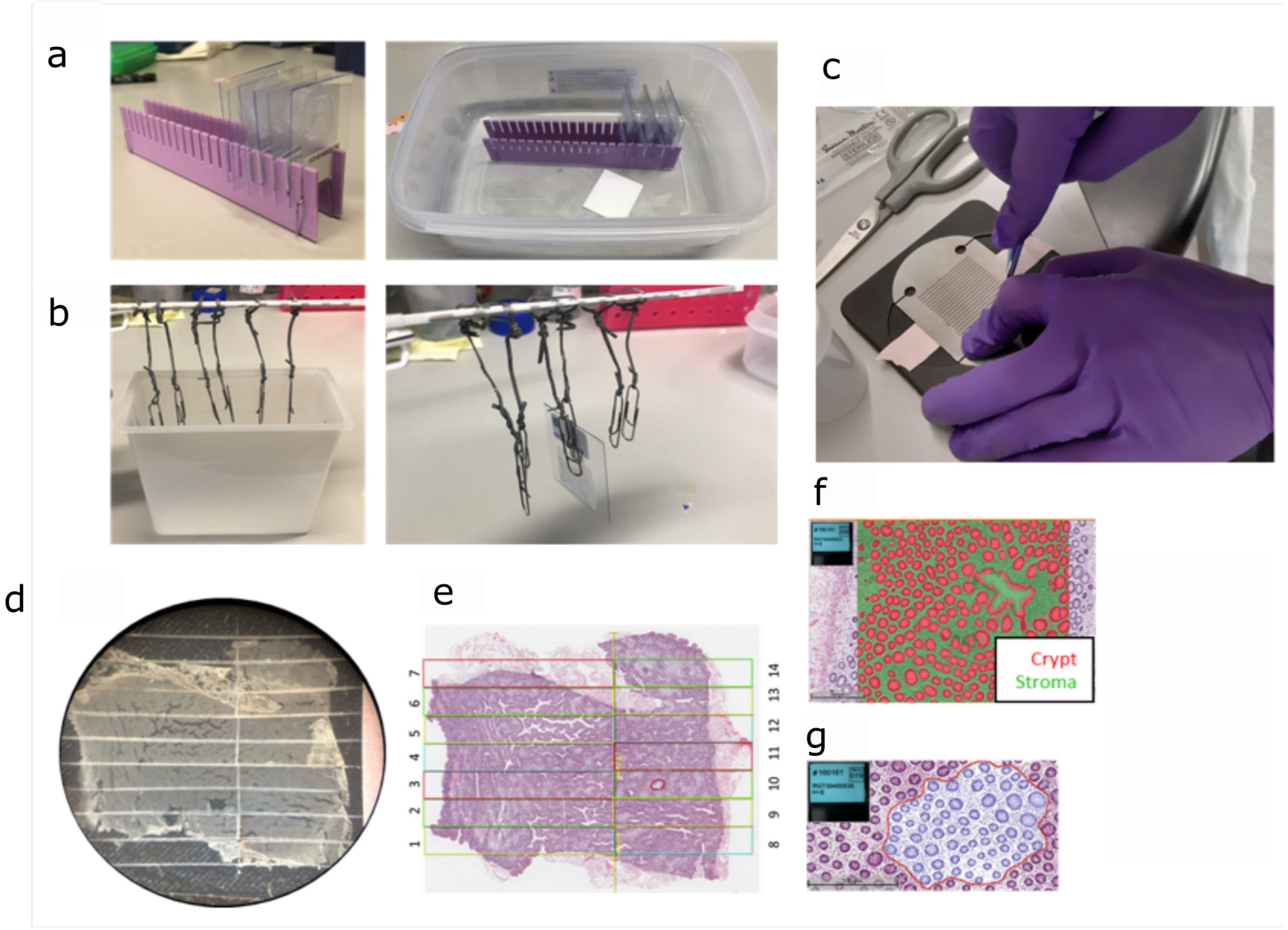

Supplementary Figure 6: AceStrip-seq sample preparation and annotation. (a) Rack for storing acetate sheets. Made in house. (b) Rack used for dewaxing. Made in house. (c) Sandwich of plastic platform, acetate section and metal stencil containing guiding slots with centres separated by 2mm. Strips were cut using scalpel. (d) Example of acetate sheet with strips. (e) H&E serial section with annotated strips on Halo. (f) Halo random forest classifier separating crypt and stroma. (g) Halo Multiplex IHC algorithm counting nuclei in crypts used to estimate crypt count per strip**.**

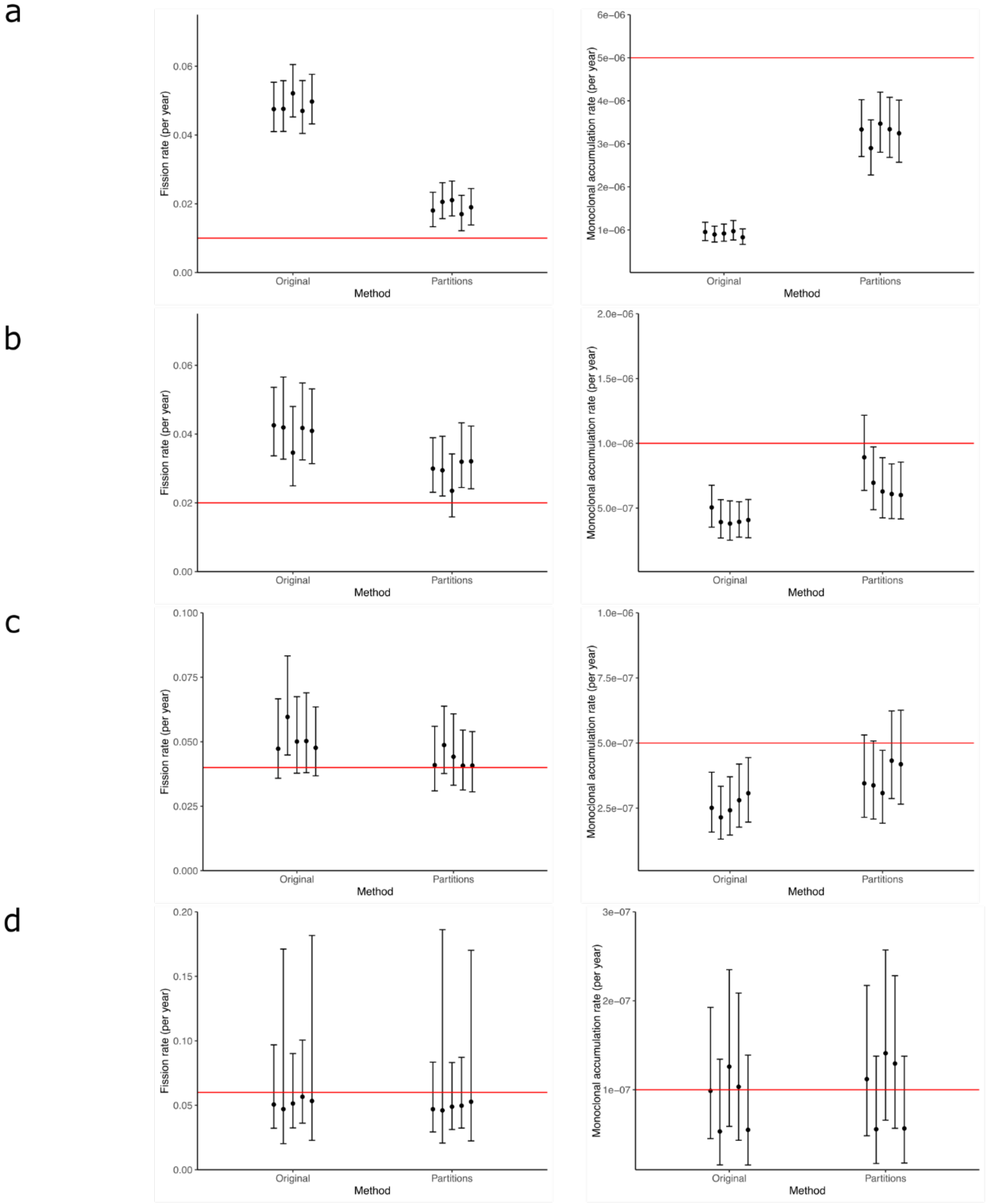

Supplementary Figure 7. Comparison of clonal dynamics inferred from simulated VAFs for a range of fixation and fission rates using the original and partitions-based statistical model described in Nicholson et al. (2018) and this study, respectively. Independent stochastic simulations of clonal induction and expansion in N=100 samples were repeated five times for each pair of baseline fixation and fission rates, which were accurately reproduced using simulated clone sizes as input for RHClones (https://github.com/ElEd2/RHClones). Simulated clone sizes were then used to generate simulated sequencing data at a range of depths and using a binomial model for alternate sequencing reads based and total number of mutated crypts in each sample. These data were in turn used to infer effective fixation and fission rates using both statistical models (black dots with 95% CIs), and estimates are compared to baseline values (red lines) ranging from extremes of high fixation with low fission to low fixation with high fission: (a) $\Delta Cfix = 5 \times 10{-}6$ $\rho = 0.01$; (b) $\Delta Cfix = 1 \times 10{-}6$ $\rho = 0.02$; (c) $\Delta Cfix = 5 \times 10{-}7$ $\rho = 0.04$; and (d) $\Delta Cfix = 1 \times 10{-}7$ $\rho = 0.06$. Source data are provided as source data file.

Supplementary Table 1: Adjacent mutations counted as clones

Sample	pos	ref	alt	Gene	AA_change	Scale
40C	153249385	G	A	FBXW7	R465C	Micro
40D	153249385	G	A	FBXW7	R465C	Micro
47D	153249385	G	A	FBXW7	R465C	Micro
47E	153249385	G	A	FBXW7	R465C	Micro
68A	7578245	G	A	TP53	R202C	Micro
68C	7578245	G	A	TP53	R202C	Micro
57G	112175411	G	T	APC	E1374*	Micro
57K	112175411	G	T	APC	E1374*	Micro
RGT00456034-5	153249385	G	A	FBXW7	R465C	AceStrip
RGT00456034-6	153249385	G	A	FBXW7	R465C	AceStrip
RGT00448542-16	7577163	G	T	TP53	0	AceStrip
RGT00448542-17	7577163	G	T	TP53	0	AceStrip
RGT00448542-13	7577157	T	A	TP53	0	AceStrip
RGT00448542-14	7577157	T	A	TP53	0	AceStrip
RGT00448542-2	7577539	G	A	TP53	R248W	AceStrip
RGT00448542-9	7577539	G	A	TP53	R248W	AceStrip
RGT00448542-17	7578388	C	T	TP53	R181H	AceStrip
RGT00448542-8	7578388	C	T	TP53	R181H	AceStrip
RGT00358732-5	7578407	G	A	TP53	R175C	AceStrip
RGT00358732-11	7578407	G	A	TP53	R175C	AceStrip
RGT00358732-12	7578245	G	A	TP53	R202C	AceStrip
RGT00358732-7	7578245	G	A	TP53	R202C	AceStrip
RGT00445817-1	7578388	C	T	TP53	R181H	AceStrip
RGT00445817-19	7578388	C	T	TP53	R181H	AceStrip
RGT00456034-7	7578262	C	T	TP53	R196Q	AceStrip
RGT00456034-8	7578262	C	T	TP53	R196Q	AceStrip
CRU00253208-18	7578210	T	C	TP53	R213	AceStrip
CRU00253208-19	7578210	T	C	TP53	R213	AceStrip
CRU00253208-9	7578210	T	C	TP53	R213	AceStrip
RGT00457316-12	7577538	C	T	TP53	R248Q	AceStrip
RGT00457316-4	7577538	C	T	TP53	R248Q	AceStrip
RGT00506490-11	7577090	C	T	TP53	R283H	AceStrip
RGT00506490-12	7577090	C	T	TP53	R283H	AceStrip
RGT00448542-16	25398284	C	A	KRAS	G12V	AceStrip
RGT00448542-17	25398284	C	A	KRAS	G12V	AceStrip
RGT00457306-13	25398284	C	A	KRAS	G12V	AceStrip
RGT00457306-14	25398284	C	A	KRAS	G12V	AceStrip
RGT00455251-9	25398285	C	A	KRAS	G12C	AceStrip
RGT00455251-10	25398285	C	A	KRAS	G12C	AceStrip

Supplementary Table 2: Mutation co-occurrence in Micro-seq

Sample	Mutation 1 (%VAF)	Mutation 2 (%VAF)
RGT00399307-71B	FBXW7 H470D (0.15%)	TP53 R248W (0.6%)
RGT00360971-74C	FBXW7 R465C (0.5%)	APC R876* (0.4%)
CRU00247491-67K	FBXW7 R465C (0.7%)	APC S1392* (0.5%)
CRU00246177-57G	APC E1374* (0.6)	APC E1353* (0.6%)
CRU00242752-36B	TP53 R248W (0.4%)	APC C1410* (0.33%)

Supplementary Table 3: Primer sequences

Amplicon/name	Sequence F' primer	Sequence R' primer	Product Size
FBXW7_R465_1	TCAAAGTGTGGAATGCAGAGAC	CCAACCATGACAAGATTTTCCCT	117
FBXW7_R465_2	TCTACAGATCGGACACTCAAAGT	AAGGGCCCAAATTCACCAA	169
APC_R876_1	GAGAGAACGCGGAATTGGTC	ATGACTTTGGCAATCTGGGC	114
APC_R876_2	TTGGAGAGAGAACGCGGAAT	GGCTGACACTTCTTCCATGAC	135
KRAS_G12_1	TAAGGCCTGCTGAAAATGACT	ATGGTCCTGCACCAGTAATATG	168
KRAS_G12_2	GGTGGAGTATTTGATAGTGTATTAACC	TAGCTGTATCGTCAAGGCAC	159
BRAF_V600E_1	AACTCTTCATAATGCTTGCTCTGA	AACTGTTCAAACTGATGGGACC	166
KRAS_Q61_1	TCCAGACTGTGTTTCTCCCT	AAAGAAAGCCCTCCCCAGTC	153
KRAS_Q61_2	CCAGACTGTGTTTCTCCCTTC	CCTCCCCAGTCCTCATGTACT	143
TP53_R273_282_1	TTGGGAGTAGATGGAGCCTG	TGCGGAGATTCTCTTCCTCT	180
TP53_R273_282_2	GGACCTGATTTCCTTACTGCC	GCTCCCCTTTCTTGCGGA	151
TP53_R213_1	GCCCCTCCTCAGCATCTTAT	ACAACCACCCTTAACCCCTC	157
TP53_R213_2	GGCCTCTGATTCCTCACTGA	CTCCCAGAGACCCCAGTTG	172
CTNNB1_D32-S45_1	AAGCGGCTGTTAGTCACTGG	CAGGACTTGGGAGGTATCCA	134
CTNNB1_D32-S45_2	TTTGATGGAGTTGGACATGG	CCTCAGGATTGCCTTTACCA	143
KRAS_A146_1	TGTGATTTGCCTTCTAGAACAGT	TCAGTGTTACTTACCTGTCTTGTC	113
KRAS_A146_2	ACAGGCTCAGGACTTAGCAAGA	ATGATTTTGCAGAAAACAGATCTGT	113
TP53_G245-R248_1	TTGGGCCTGTGTTATCTCCT	CCAGTGTGATGATGGTGAGG	122
TP53_G245-R248_2	TGGCTCTGACTGTACCACCA	GGCTCCTGACCTGGAGTCTT	118
TP53_R175_1	TGGCCATCTACAAGCAGTCA	TCAGTGAGGAATCAGAGGCC	152
TP53_R175_2	GTGCAGCTGTGGGTTGATTC	TGCTCACCATCGCTATCTGA	140
APC_R1450_1	AAGCCCCAGTGATCTTCCAG	AGCTTGCTTAGGTCCACTCT	157
APC_R1450_2	GCAGTGGAATGGTAAGTGGC	TGCTTAGGTCCACTCTCTCTC	178
CS1/CS2 (added at the start of F or R primer)	ACACTGACGACATGGTTCTACA	TACGGTAGCAGAGACTTGGTCT	

Supplementary Table 4: Multiplex groups

Multiplex group	Amplicon	Volume in total of 800 µl water
MP1	K_Q61_1	2
MP1	P_R273_282_1	2
MP1	APC_1397_1429_1	2
MP2	K_Q61_2	2
MP2	P_R213_2	2
MP2	APC_1397_1429_2	2
MP3	P_R273_282_1	2
MP3	P_R213_1	2
MP3	CTNNB1_1	2
MP4	P_R273_282_2	2
MP4	CTNNB1_2	2
MP4	APC_1338_1378_2	2
MP5	K_A146_1	2
MP5	APC_1450_1	2
MP5	APC_1338_1378_1	2
MP6	P_R175_2	2
MP6	APC_1450_2	2
MP6	APC_1338_1378_2	2
MP7	P_G245_248_1	2
MP7	APC_554_564_1	2
MP7	APC_1338_1378_1	2
MP8	P_R175_1	2
MP8	APC_554_564_1	2
MP8	APC_1338_1378_1	2
MP9	K_A146_2	2
MP9	P_G245_248_2	2
MP9	P_R175_2	2
MP10	F_R465_1	2
MP10	Braf_1	2
MP10	APC_1397_1429_1	2
MP11	A_R876_1	2
MP11	KRAS_G12_1	2
MP11	APC_1294_1328_1	2
MP12	F_R465_2	2
MP12	A_R876_2	1
MP12	KRAS_G12_2	4
MP12	APC_1294_1328_2	2

Supplementary Table 5: PCR components

Component	Volume (µl)
Primer mix (1 µM)	5
dNTP (10 mM- New England BioLabs)	0.5
5X Phusion HF Reaction Buffer	5
Nuclease free H2O (Ambion)	13.25/12.25
Phusion High Fidelity DNA Polymerase (New England Biolabs)	0.25
Genomic DNA	1 (WS-DNA)/2 (LCM and STRP-Seq)
Total	25
