# Supplementary material for: Clonal biases dictate availability of colonic cancer driver mutations for transformation
Source: Nat Commun. 2026 Apr 16;17:5295. doi: 10.1038/s41467-026-71944-5 (PMC13269798; doi:10.1038/s41467-026-71944-5)
Supplement: Supplementary file 2 — Reporting Summary [file 41467_2026_71944_MOESM2_ESM.pdf]

nature portfolio

Corresponding author(s): Douglas Winton

Last updated by author(s): Dec 2, 2025

Reporting Summary

Nature Portfolio wishes to improve the reproducibility of the work that we publish. This form provides structure for consistency and transparency in reporting. For further information on Nature Portfolio policies, see our Editorial Policies and the Editorial Policy Checklist.

Statistics

For all statistical analyses, confirm that the following items are present in the figure legend, table legend, main text, or Methods section.

n/a	Confirmed	
☐	☒	The exact sample size (*n*) for each experimental group/condition, given as a discrete number and unit of measurement
☐	☒	A statement on whether measurements were taken from distinct samples or whether the same sample was measured repeatedly
☐	☒	The statistical test(s) used AND whether they are one- or two-sided<br>*Only common tests should be described solely by name; describe more complex techniques in the Methods section.*
☐	☒	A description of all covariates tested
☐	☒	A description of any assumptions or corrections, such as tests of normality and adjustment for multiple comparisons
☐	☒	A full description of the statistical parameters including central tendency (e.g. means) or other basic estimates (e.g. regression coefficient) AND variation (e.g. standard deviation) or associated estimates of uncertainty (e.g. confidence intervals)
☐	☒	For null hypothesis testing, the test statistic (e.g. *F*, *t*, *r*) with confidence intervals, effect sizes, degrees of freedom and *P* value noted<br>*Give P values as exact values whenever suitable.*
☒	☐	For Bayesian analysis, information on the choice of priors and Markov chain Monte Carlo settings
☒	☐	For hierarchical and complex designs, identification of the appropriate level for tests and full reporting of outcomes
☐	☒	Estimates of effect sizes (e.g. Cohen's *d*, Pearson's *r*), indicating how they were calculated

Our web collection on statistics for biologists contains articles on many of the points above.

Software and code

Policy information about availability of computer code

Data collection	No software was used.
Data analysis	Data analysis was performed with R (version 4.4.0). Spatial Experiment package was used for Visium Data Analysis and is described in Methods (version 1.18.1). Mathematical modeling is described in Methods. Code is available on GitLab. https://gitlab.com/davidtourigny/crypt-clone-dynamics.

For manuscripts utilizing custom algorithms or software that are central to the research but not yet described in published literature, software must be made available to editors and reviewers. We strongly encourage code deposition in a community repository (e.g. GitHub). See the Nature Portfolio guidelines for submitting code & software for further information.

Data

Policy information about availability of data

All manuscripts must include a data availability statement. This statement should provide the following information, where applicable:

- Accession codes, unique identifiers, or web links for publicly available datasets
- A description of any restrictions on data availability
- For clinical datasets or third party data, please ensure that the statement adheres to our policy

The raw DNA amplicon-seq data generated in this study have been deposited in the NCBI database under accession code PRJNA1372027 [https://

www.ncbi.nlm.nih.gov/bioproject/PRJNA1372027].

The raw Spatial transcriptomics data generated in this study have been deposited in the GEO database under accession code GSE312204 [https://www.ncbi.nlm.nih.gov/geo/query/acc.cgi?acc=GSE312204].

Processed data are available as supplementary data. Source data are provided with this paper.

Research involving human participants, their data, or biological material

Policy information about studies with human participants or human data. See also policy information about sex, gender (identity/presentation), and sexual orientation and race, ethnicity and racism.

Reporting on sex and gender	Informed consent was obtained from each participant. Source data provided for cohort information includes patient sex. An equivalent number of male (n=115, mean age =69) and female (n=106, mean age = 67) participants were included in the study. The overall patient cohort was on purposely gender balanced with a wide range of ages included. Patient consent has been obtained for publishing indirect identifiers, including gender and age.
Reporting on race, ethnicity, or other socially relevant groupings	No variable related to race, ethnicity, or other socially relevant grouping have been used in the present study.
Population characteristics	Normal colon tissue samples were obtained from 221 patients undergoing colorectal surgery. Age ranged from 33-91 (mean age 68). Detailed information can be found in Supplementary Table 1.
Recruitment	This is a retrospective study. Colon tissue samples were obtained from patients undergoing colorectal surgery.
Ethics oversight	Samples were collected from 2 sites in UK, Addenbrooke's Hospital Cambridge and St James University Hospital Leeds. This study was performed under full local research ethical committee approval (REC 15/WA/0131, 06/Q0108/307, 17/EE/0265 and 12/LO/1217) according to UK Home Office regulations, with informed consent obtained from all participants. Wales (REC7) and London Bloomsbury Research Ethics Committees approved this study.

Note that full information on the approval of the study protocol must also be provided in the manuscript.

Field-specific reporting

Please select the one below that is the best fit for your research. If you are not sure, read the appropriate sections before making your selection.

☒ Life sciences ☐ Behavioural & social sciences ☐ Ecological, evolutionary & environmental sciences

For a reference copy of the document with all sections, see nature.com/documents/nr-reporting-summary-flat.pdf

Life sciences study design

All studies must disclose on these points even when the disclosure is negative.

Sample size	This is a descriptive retrospective study, thus no sample size calculation was performed. Sample size was determined by the availability of the data.
Data exclusions	No data were excluded from the analysis.
Replication	Even though our results come from 2 cohorts (Addenbrookes and Leeds Hospitals, UK), the data were pooled together to increase sample number.
Randomization	As described above, this is a retrospective study; patients were not allocated to groups.
Blinding	Mutation calling was performed blinded using fluidigm barcode numbers.

Reporting for specific materials, systems and methods

We require information from authors about some types of materials, experimental systems and methods used in many studies. Here, indicate whether each material, system or method listed is relevant to your study. If you are not sure if a list item applies to your research, read the appropriate section before selecting a response.

Materials & experimental systems

n/a	Involved in the study
☐	☒ Antibodies
☒	☐ Eukaryotic cell lines
☒	☐ Palaeontology and archaeology
☒	☐ Animals and other organisms
☒	☐ Clinical data
☒	☐ Dual use research of concern
☒	☐ Plants

Methods

n/a	Involved in the study
☒	☐ ChIP-seq
☒	☐ Flow cytometry
☒	☐ MRI-based neuroimaging

Antibodies

Antibodies used

REG4 (HPA, HPA046555, Polyclonal, 1:1000), MUC5AC (Abacm, ab198294, EPR16904, 1:200) and CDX2 (Sigma, AMAB91828, CL12974, 1:500) .

Validation

REG4 antibody has been validated by Human Protein Atlas. MUC5AC antibody has been validated by Abcam and has been cited in more than 10 publications CDX2 antibody has been validated by Sigma using orthogonal RNAseq.

Plants

Seed stocks

Report on the source of all seed stocks or other plant material used. If applicable, state the seed stock centre and catalogue number. If plant specimens were collected from the field, describe the collection location, date and sampling procedures.

Novel plant genotypes

Describe the methods by which all novel plant genotypes were produced. This includes those generated by transgenic approaches, gene editing, chemical/radiation-based mutagenesis and hybridization. For transgenic lines, describe the transformation method, the number of independent lines analyzed and the generation upon which experiments were performed. For gene-edited lines, describe the editor used, the endogenous sequence targeted for editing, the targeting guide RNA sequence (if applicable) and how the editor was applied.

Authentication

Describe any authentication procedures for each seed stock used or novel genotype generated. Describe any experiments used to assess the effect of a mutation and, where applicable, how potential secondary effects (e.g. second site T-DNA insertions, mosiacism, off-target gene editing) were examined.
