# Supplementary material for: Clonal biases dictate availability of colonic cancer driver mutations for transformation
Source: Nat Commun. 2026 Apr 16;17:5295. doi: 10.1038/s41467-026-71944-5 (PMC13269798; doi:10.1038/s41467-026-71944-5)
Supplement: Supplementary file 3 — Transparent Peer Review File [file 41467_2026_71944_MOESM3_ESM.pdf]

nature portfolio

Peer Review File

Clonal biases dictate availability of colonic cancer driver mutations for transformation

Corresponding Author: Dr Douglas Winton

This file contains all reviewer reports in order by version, followed by all author rebuttals in order by version.

Version 0:

Reviewer comments:

Reviewer #1

(Remarks to the Author)
The initial stages of colon cancer are poorly understood, with most insights being generated using genetic mouse models. In the current manuscript, the authors explore the presence of common driver mutation in human colon as first signs of pre-malignant lesions with apparent normal phenotype. The manuscript starts with a systematic investigation using micro-seq where semi-bulk pathches of FFPE sectioned materal (totaling approximately 200 crypts) are being sequenced using a targeted amplicon panel. Analysis of deep-sequenced genome fragments enables the identification of minor variation in nucleotide calling, suggesting the presence of a mutant clone.
First, the pattern and nature of the found mutations are compared to the current reference dataset of Lee-Six., validating the current methodology that has the advantage of higher throughput and thus the potential to pick up rare mutational events. Indeed, the micro-seq analysis identified the presence of most common driver mutations in normal human colon. Next, it was inferred that oncogenic mutation in FBXW7 and KRAS are positively selected, in contract to truncating mutations in for example APC or TP53. To infer the nature of positive selection on both oncogenes, modelling and mathematics are applied which suggest that FBXW7 has increased fixation within a crypt, while KRAS manifests elevated fission rates.
1) Unfortunately, both predicted phenotypes are not experimentally demonstrated.

Next, it is stated that three driver mutations are required for cancers to develop. Using the assessed mutation frequencies and their clonal dynamics, it is modelled how three mutations can co-occur in the same cell. Since no positive selection is measured on truncating mutations in APC or TP53 (rendering these tumor suppressors heterozygous), it is assumed that both mutations emerge by de novo mutagenesis. As such, a major impact for tripartite co-occurrence is placed on the positive selected KRAS or FBXW7 mutations.
I may not have understood the matter, but an unknown variable is the behavior of a full APC (or TP53) deficient clone. While truncating mutations in both alleles may very well be the result of de novo mutagenesis, once an APC deficient clone emerges it may experience such growth advantages that it will dwarf the positive effects on KRAS and/or FBXW7.
2) To improve insights into the behavior of de novo APC deficient clones, similar micro-seq analysis is warranted on colon material from FAP patients. While the homeostatic background is APC heterozygous, it is to be anticipated that APC deficient clones are readily detectable. Moreover, a) it is of interest to investigate the phenotype of APCnull patches and whether some may retain a normal appearance, and b) APC null patches may be identified with a secondary mutation.

Ultimately, this manuscript provides a systematic analysis on the presence of cancer driver mutations in normal human colon. While it provides a thorough documentation, the fact that such mutations are identified is not surprising. Moreover, the insights into the behavior and consequences of the mutational frequencies is inferred by abstract mathematics and modelling that feels indirect without experimental validation or mechanistic follow-up.

A strategy to boost scientific interest may be to map the mutational presence in chronically inflamed mucosa, aforementioned FAP patients, or other high-risk scenario's.

Minor:
How does the no phenotypic APC truncations in human relate to mouse studies with apparent selective advantage for the heterozygous situation? (Vermeulen 2013)

KRAS Q61 is extremely rare in colon cancer. Its relative abundant identification in normal human colon suggests a potential

mismatch with its potential to drive cancer development. Is its deviating frequency in relation to colon cancer a possibility within statistical margins, or is there hidden biology?

KRAS mutations were called in 80% of the REG4-high regions. Were these regions also analyzed for mutations in NRAS or BRAF to explain the other 20%?

Regarding the spatial transcriptomic dataset. I may understand that subtle phenotypes for heterozygous tumor suppressors is challenging to pick up. Is the potential clone size of FBXW7 sufficient to be picked up, in relation to KRAS mutant clones that expand in size and elevate their relative contribution to the semi-bulk RNA profile?

(Remarks on code availability)

Reviewer #2

(Remarks to the Author)
We received the manuscript entitled, “Clonal biases dictate availability of colonic cancer driver mutations for transformation”, with substantial enthusiasm. Our opinion is of that the study addresses an important and timely topic in the field – of the clonal dynamics of driver mutations that exist in phenotypically normal tissues. The authors utilize sequencing technologies on a cohort of human colonic specimens along with computational modeling to differentiate models of clonal expansion (fixation – monoclonal crypt conversion vs. fission resulting in increased patch size). The study was well done and was able to differentiate these two models based on mutational patterns in different oncogenes. The KRAS result has been reported in a previous study (Olpe et al., 2021). There are areas in the paper that the authors should address to enhance clarity.
Comments:
1. The authors utilize 3 resolutions of targeted amplicon sequencing, micro-seq (all the way down to crypt level), aceStrip-seq (tissue strips), and Section-seq (bulk). The authors should clarify how those technologies are used. Right now, it seems like micro-seq is predominantly used.
2. For micro-seq, the authors should also clarify if single crypts are sequenced like previous studies. For some of the results, it seems like multiple crypts are grouped together and sequenced.
3. As above only 285/76800 clones were determined to have mutations, yet ~41% APC mutations and 18% KRAS mutations were detected. These drivers were not detected in previous studies; please reconcile these discrepancies (i.e. single vs multi crypts sequenced for example).
4. Most of the images provided were tissue sections of colonic crypts en face. Can you comment on the artifacts produced given the crypts are not sampled cross-sectionally. I.e. especially for the fixation model, that you are not sampled the top and bottom of the crypts entirely?
5. A limitation in the study is that mutations in only 5 genes were profiled. The mutation statuses of many genes are unknown, and it is very likely that there are interactions amongst mutations that give rise to phenotypes that are missed in the study. A point I would like to make about this is the phenotypic heterogeneity conferred by KRAS mutations (Fig. 4h), which may have arisen due to other mutations occurring in the tissue. Specifically, what about the ORDER of mutations between Apc-KrasG12 compared to KrasG12-Apc? Do these scenarios give different phenotypes, such as one giving rise to the hyperplastic polyp phenotype? The order can probably be deciphered by looking at VAF differences between the two mutations.
6. The nuisances and assumptions of the computational model are also quite limited (for example, assuming independence of mutations and independence of monoclonal conversion and fission). It would be helpful to biologists to explain the model better in terms of how the two models are independent (one would that both crypt fission and fixation depends on biased proliferation). Also, it would seem like fixation is a rather limited form of clonal expansion since it will only be limited to a single crypt, while fission will generate patches of monoclonal crypts. How the model of clonal dynamics from individual patients is defined and related to “lifetime risk of developing cancer” is also not clear.
7. The manuscript focuses a lot on KRAS mutations and not so much on FBXW7. This is due to regions with KRAS mutations exhibiting very different transcriptomic phenotypes compared to the rest of the conditions. However, KRAS mutation confers secretory differentiation, and thus those cells become distinct from colonocytes. This aspect of RAS-MEK-ERK signaling has been known historically (Heuberger et al., 2014). We wonder if this substantial difference in differentiation is masking more subtle effects of the other mutations (such as haploinsufficient effects of loss of 1 allele of APC)
8. Are biallelic loss of APC function detected in “normal” colon?
9. From Simon Leedlam’s work, there are defined signatures for stem-like tumor cells and fetal-like tumor cells (or adenoma vs serrated cells). This current paper tarnscriptomically examined the fetal/serrated/secretory signatures. We are wondering if the authors can also query the stem signatures.
10. Regarding lineage confusion, one really needs to demonstrate that cells are lineage confused at the single cell level (i.e. both signatures admixed in one cell). Right now, it seems like a region is lineage confused but the signatures are expressed in different parts of the region (in different cells). Also, even if there are cells that expressed mixed signatures, this may be a transitory state (one state on its way of converting into another state) and not a permanent mixed identity, and thus, cannot be formally defined as lineage confusion.
Minor:
Figure 4E needs a randomized control.

(Remarks on code availability)

Version 2:

Reviewer comments:

Reviewer #1

(Remarks to the Author)
Skoufou-Papoutsaki and colleagues responded very eloquently to the concerns that I raised. In parallel, while addressing the comments, the manuscript now contains large parts that are being rewritten both textual and conceptual.

Generally, the topic of interest, being presence and behavior of mutant clones in otherwise normal colon epithelium of patients, is highly relevant to understand the first stages of tumorigenesis, the relative burden of the disease during aging, and to start understanding why the onset age keeps getting younger. The revised manuscript is improved in terms of readability and flow due to the textual changes (like abstract and intro) that now explicitly state the knowledge gaps/rationale, research questions and unique approach. Moreover, comments have been addressed including new analyses, that subsequently have been incorporated into the revised text.

I thank the authors for their work on a highly relevant and interesting topic.

(Remarks on code availability)

Reviewer #2

(Remarks to the Author)
The authors did a great job addressing our comments.

(Remarks on code availability)

Open Access This Peer Review File is licensed under a Creative Commons Attribution 4.0 International License, which permits use, sharing, adaptation, distribution and reproduction in any medium or format, as long as you give appropriate credit to the original author(s) and the source, provide a link to the Creative Commons license, and indicate if changes were made.
In cases where reviewers are anonymous, credit should be given to 'Anonymous Referee' and the source.
The images or other third party material in this Peer Review File are included in the article's Creative Commons license, unless indicated otherwise in a credit line to the material. If material is not included in the article's Creative Commons license and your intended use is not permitted by statutory regulation or exceeds the permitted use, you will need to obtain permission directly from the copyright holder.

To view a copy of this license, visit https://creativecommons.org/licenses/by/4.0/

REVIEWER COMMENTS

Reviewer #1 (Remarks to the Author): Expertise in CRC genomics and phenotypic heterogeneity/plasticity

The initial stages of colon cancer are poorly understood, with most insights being generated using genetic mouse models. In the current manuscript, the authors explore the presence of common driver mutation in human colon as first signs of pre-malignant lesions with apparent normal phenotype. The manuscript starts with a systematic investigation using micro-seq where semi-bulk patches of FFPE sectioned materal (totaling approximately 200 crypts) are being sequenced using a targeted amplicon panel. Analysis of deep-sequenced genome fragments enables the identification of minor variation in nucleotide calling, suggesting the presence of a mutant clone.
First, the pattern and nature of the found mutations are compared to the current reference dataset of Lee-Six., validating the current methodology that has the advantage of higher throughput and thus the potential to pick up rare mutational events. Indeed, the micro-seq analysis identified the presence of most common driver mutations in normal human colon. Next, it was inferred that oncogenic mutation in FBXW7 and KRAS are positively selected, in contract to truncating mutations in for example APC or TP53. To infer the nature of positive selection on both oncogenes, modelling and mathematics are applied which suggest that FBXW7 has increased fixation within a crypt, while KRAS manifests elevated fission rates.
1) Unfortunately, both predicted phenotypes are not experimentally demonstrated.

The Reviewer is correct that the predicted phenotypes are an interpretation of experimental data. Interpreting variant allele frequencies (VAFs) in terms of clone size is relatively standard practice in cancer biology, and over-representation of a mutation by comparison to matching control sequences is an established method used to infer positive selection (formalised using dN/dS and related methods, (Lee-Six *et al*, 2019; Gerstung *et al*, 2020; Sottoriva *et al*, 2015; Martincorena *et al*, 2017a). Interpretations of sequencing data are commonly viewed as sufficient to serve these purposes. We accept that an additional element covered in the manuscript is to determine how positive selection is mediated in the context of the normal glandular structure comprising the colonic epithelium. However, our distinction between two modes of selection (fixation and fission) merely serves to formalise two self-evident phases in its execution – that mutant cells must initially compete within a gland and once that process is complete then mutant glands can further expand due to acquired behaviours.

Our approach developed over many years has been to be minimalist in our assumptions about how selection is mediated. That is, we have devised strategies to measure and compare changes in clonal competition between clonogenic cells

within individual intestinal glands that are conferred by different somatic events and different acquired behaviours. One challenge in deriving metrics that apply to all gene mutations that are inherently different in the pathway and processes they regulate is to be able to directly compare biases irrespective of how they are mediated e.g. whether they promote proliferation or impact apoptosis and survival. The second challenge has arisen from the acceptance in the stem cell field that self-renewal is not just a function of bone fide stem cells but is also a result of extensive plasticity of their descendants such that they can contribute to intragland competition- a behaviour that may be altered by oncogenic drivers. Essentially our approach is to integrate all these variables into a simple model by determining the outcomes of clonal competition in terms of the frequency of variants surviving, their occupancy of the crypt epithelium, and subsequent expansion by the known process of fission.

In sum our response is that we have interpreted mutation counts and VAF values in a way that is agnostic to different biological processes that mediate clonal competition. That selection occurs for *FBXW7* and *KRAS* missense mutations is a result of the dN/dS analysis directly applied to DNA sequencing data, an analysis that has been used many times before in the mutational landscape field (Cagan *et al*, 2022; Lawson *et al*, 2025; Martincorena *et al*, 2017). Moreover, our statistical inference model for clone dynamics is highly conservative in assigning intraglandular fixation and later crypt fission biases. Using a parsimonious Bayesian approach, our model seeks to identify the posterior distributions over fixation and fission rates that are most compatible with these observed experimental data. We have reported biased behaviour as that which departs from neutral clone dynamics (accessed by internal controls with synonymous mutations) and attributes an increased fixation probability and fission rate to FBXW7 R465C and KRAS G12 missense mutations, respectively, with a high degree of certainty. These selection biases are therefore entirely consistent with the results of dN/dS and validated experimental with an immunohistochemical marker (REG4) in the case of KRAS. What we have certainly not attempted to propose is any underlying biological mechanism linking these selection biases to the mutations, which the Reviewer is correct would require additional experimental validation beyond the scope of this paper.

Next, it is stated that three driver mutations are required for cancers to develop. Using the assessed mutation frequencies and their clonal dynamics, it is modelled how three mutations can co-occur in the same cell. Since no positive selection is measured on truncating mutations in APC or TP53 (rendering these tumor suppressors heterozygous), it is assumed that both mutations emerge by de novo mutagenesis. As such, a major impact for tripartite co-occurrence is placed on the positive selected KRAS or FBXW7 mutations.
I may not have understood the matter, but an unknown variable is the behavior of a

full APC (or TP53) deficient clone. While truncating mutations in both alleles may very well be the result of de novo mutagenesis, once an APC deficient clone emerges it may experience such growth advantages that it will dwarf the positive effects on KRAS and/or FBXW7.
2) To improve insights into the behavior of de novo APC deficient clones, similar micro-seq analysis is warranted on colon material from FAP patients. While the homeostatic background is APC heterozygous, it is to be anticipated that APC deficient clones are readily detectable. Moreover, a) it is of interest to investigate the phenotype of APCnull patches and whether some may retain a normal appearance, and b) APC null patches may be identified with a secondary mutation.

Thank you. The Reviewer's appreciation of the issues impacting on this analysis is correct and highlights a shortcoming of our original approach. In attempting to be minimalist in extrapolating from our experimental observations we modelled only the specific mutations detected to predict how often these would be expected to co-occur in individual crypts assuming they were independent events. These minimalist predictions of co-occurrence frequencies were compared to the frequency of CRCs with the same combination of events. This analysis suggested that three-hit models for CRC development are possible but only under conditions where the mutations are not positively selected, acknowledging the impact of selection would require a revision of the three-hit model. However, we accept this restricted our analysis and excluded other realistic scenarios for CRC development including the potential for strong positive selection following a second hit in APC as proposed by the Reviewer.

In deciding how best to respond to the Reviewer's comment, and in particular the significant potential challenges of repeating our analyses in FAP patients without having immediate access to the relevant tissues, we became aware of a recent detailed online report, published since our original submission, that has essentially performed a very similar analysis to that suggested by the Reviewer (Stratton *et al*, 2025). Sequencing of 110 normal appearing crypts in FAP patients identified only one that potentially had a second hit in addition to the germline mutation (though this may have been in *cis* to that latter allele). As expected, adenomatous polyps and aberrant crypt foci did commonly have second sporadic mutations. However, while clarifying these points raised by the Reviewer an unresolved issue is whether crypts deficient in APC due to mutation of both alleles are immediately positively biased. Indeed, available evidence from mouse studies suggest that a critical density of APC mutant crypts is required with reduced densities abrogating, and aggregation of mutant colonic crypts augmenting, adenoma formation (Fischer *et al*, 2014; Gaynor *et al*, 2025).

Thus, the extent and timing of positive selection conferred by APC deficiency remains unknown and is an extremely challenging question to address experimentally. Consequently, we decided to modify our original analysis by adapting an established simulation model that incorporates multiple hits of APC and other tumour suppressor genes, and was originally parameterised with fixation and fission

rates (including those for biallelic APC inactivation) in a way suggested to reproduce CRC incidence rates (Paterson *et al*, 2020). We refined this original model by using our own inferred clone dynamics for the monoallelic mutations observed and considered two extreme scenarios for the unknown impact of biallelic APC inactivation. The first scenario assumes fixation and fission rate biases that are found to heavily outweigh the inferred biases of KRAS activation (4-5 fold higher), as suggested by the Reviewer and assumed by the authors of the original model (Paterson *et al*, 2020). In the second scenario, no additional increase in fixation or fission rates were assumed beyond that measured for monoallelic APC inactivation. We followed the same rationale of the original study to compare predictions of the simulation model, given in terms of probabilities of finding a crypt with a given combination of mutations by a given age, with observed incidence rates of CRCs harbouring those same mutations.

This analysis suggested—as the Reviewer suspected-- that in the presence of a high selection bias for biallelic APC loss, crypts carrying a mutation in three different genes are more likely to originate from crypts where biallelic inactivation of APC has occurred. This should be contrasted with the case where no additional bias is conferred to APC deficient clones, where an origin from mutation in one of the other genes (TP53, KRAS) becomes more probable (see also response below to Reviewer #2's comment on mutational ordering). More notably and in direct response to the Reviewer's point, the results also support the claim that positive biases (seen now with APC deficiency and previously demonstrated with KRAS activation) are not consistent with three-hit models of CRC. This non-intuitive, but insightful, conclusion arises from the fact that stronger positive biases are predicted to generate many more crypts with three or more driver events and these far outnumber the observed frequency of CRCs with the same events. Therefore, additional hits are required to explain the observed rates of transformation when selection in normal tissue is taken into account (see also the recent preprint (Cheek *et al*, 2025)that extends this principle to other tissue types). The formal treatment of this topic has highlighted this effect and to a significant degree has altered the focus of our narrative and we thank the Reviewer for promoting this outcome by their comment.

The associated changed text and new results sections are highlighted in the manuscript (lines 246-287 and see also Fig. 3c-d in the revised manuscript).

Ultimately, this manuscript provides a systematic analysis on the presence of cancer driver mutations in normal human colon. While it provides a thorough documentation, the fact that such mutations are identified is not surprising. Moreover, the insights into the behavior and consequences of the mutational frequencies is inferred by abstract mathematics and modelling that feels indirect without experimental validation or mechanistic follow-up.

We entirely agree about the expectation that powerful mutational processes acting in the colonic epithelium will generate cancer driver mutations. However, the extent to which selection biases for mutations in normal tissue explains their representation in CRC remains unknown (see above response to this Reviewer's previous point). Our prior demonstration that activating mutations of KRAS possess such biases provided partial motivation for a wider screen of CRC drivers. An additional motivation was that the initial effort to detect such events was itself surprising in not detecting mutations of the most common CRC driver events, TP53 and APC, despite identifying many less common drivers that in total affected 1% of all crypts in normal epithelium of aged individuals (Lee-Six et al., 2019).

As regards the abstract nature of our study we reference our comments on the strategy above, and our choice to adopt and extend an established and previously published mathematical model (Paterson *et al*, 2020) in the revised manuscript in favour of the one presented in the original version.

A strategy to boost scientific interest may be to map the mutational presence in chronically inflamed mucosa, aforementioned FAP patients, or other high-risk scenario's.

The relevant FAP results from the study published online (Stratton *et al*, 2025) is now referenced in the manuscript (lines 436-441 in the revised manuscript). More generally, as regards to other risk scenarios we note that colonic tissue lacking overt pathologies has a wide range of what would be classified as normal phenotypic variation. While we would suggest that a full analysis of chronic inflamed mucosa was beyond the scope of the current study, which was designed to specifically address the accumulation and expansion of driver clones in non-diseased tissue, we agree with the reviewer that it would be interesting to relate the type of mutations found based on some relevant histological information. Consequently, we performed histological assessment of all our patient samples with the help of a colorectal pathologist and stratified them based on morphological severity, either minor inflammation or hyperplastic changes that would indicate proximity to a tumour location with the patient history and clinical data also considered. Patient samples were classified as normal (morphology group 1), hyperplastic changes without inflammation (morphology group 2), minor inflammation, all focal (morphology group 3), or significant inflammation (morphology group 4). None of our samples were found within morphology group 4, and we studied the distributions of all mutations and putative driver mutations (defined with boostDM) across the remaining morphology groups (Figure R1). Using the Kruskal-Wallis test, we did not observe any enrichment of mutations or driver mutations overall or for any of the four genes assessed (*APC*, *TP53*, *KRAS*, *FBXW7*) associated with one or more of the three morphology groups.

Currently, because the outcome was negative this analysis is only included here for the Reviewer's information but we would be happy to incorporate it into the manuscript if the Reviewer feels it appropriate.

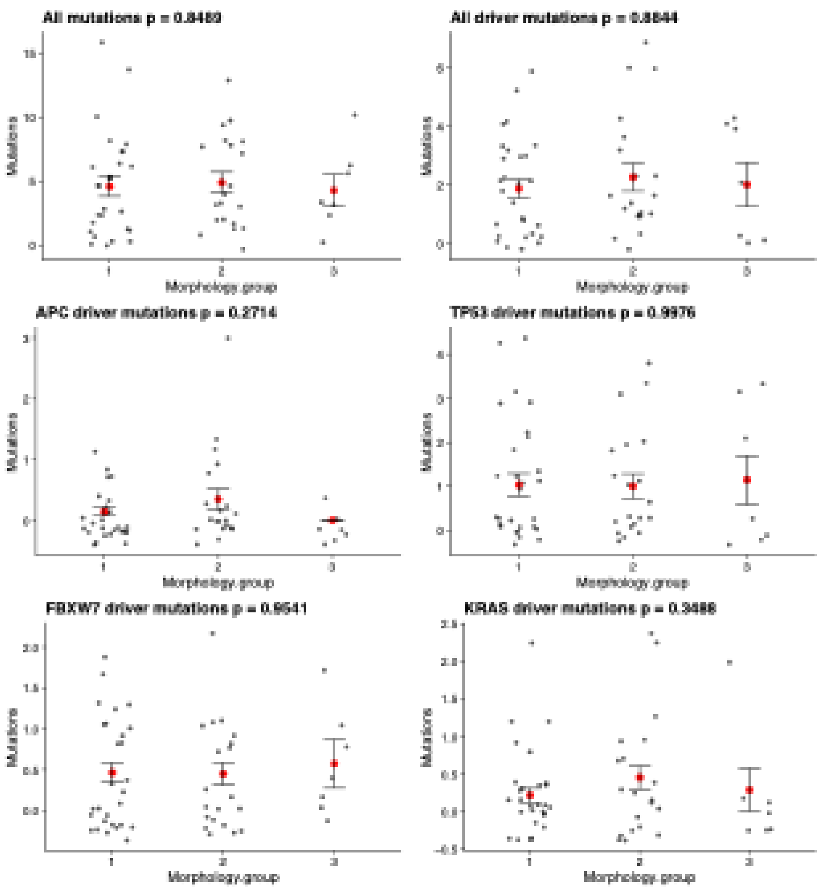

Figure R1. Mutation counts across samples stratified by morphology group. In each case, the p value is far from the decision boundary.

Minor:

How does the no phenotypic APC truncations in human relate to mouse studies with apparent selective advantage for the heterozygous situation? (Vermeulen 2013)

We are aware murine experiments suggest that (heterozygous) inactivation of a single APC allele confers a selective advantage over adjacent wild type stem cells, leading to dominance (higher fixation probability) of mutations within the crypt (see and Vermeulen et al., 2013, as the Reviewer points out). However, other studies show that unlike, biallelic ACP inactivation, there is no *fission* (expansion) bias for monoallelic APC inactivation (Fisher et al., 2012). Our results on fission rate inferences are therefore consistent with previous observations. On the other hand, we point out that detecting and quantifying *fixation* biases using our method relies on a comparison of inferred fixation rates for specific point mutations to other (e.g. synonymous) mutations within the same trinucleotide context (as we did for FBXW7

R465C). Due to a paucity of experimental observations in this case, the same approach did not enable us to attribute a positive fixation bias to any of the APC truncating mutations with an appropriate degree of confidence. Therefore, while no fixation bias was observed, we cannot rule out that other heterozygous APC mutations in humans confer the same selective fixation bias observed in mouse models and have updated the revised manuscript to clarify this point (lines 407-409 in the revised manuscript).

KRAS Q61 is extremely rare in colon cancer. Its relative abundant identification in normal human colon suggests a potential mismatch with its potential to drive cancer development. Is its deviating frequency in relation to colon cancer a possibility within statistical margins, or is there hidden biology?

We thank the Reviewer for raising this interesting point regarding the observation that *KRAS* mutations Q61L (and A146P) are found at high frequency in normal colon, while their detection in CRC is comparably rare next to the common *KRAS* missense variants at G12. The question: “to what extent does occurrence/selection for certain variants in normal tissue relate to their occurrence in cancer?” is of key importance to this study as well as others (see response to this Reviewer’s previous point and recent preprint by (Cheek *et al*, 2025). To expand upon this point in the case of these specific groups of *KRAS* mutations, we leveraged the approach used in our previous work (Skoufou-Papoutsaki *et al*, 2025). Here, we rationalised that, if the predicted prevalence (mutational burden) of a driver mutation in normal tissue intersects that observed in CRC within the lifetime of an individual, then the observed frequency of a mutation in cancer can be entirely explained by its clone dynamics in normal tissue alone (for example, in Skoufou-Papoutsaki et al we observe this to be the case for *STAG2*). Conversely, if there is a mismatch, presumably the scale of that mismatch reflects the potential for those mutations to augment (in case of an underestimate) or impair transformation. We therefore calculated mutational burden for these two groups of driver mutations and compared with frequencies of the respective mutations in CRC (Figure R2). As expected, the mutational burden of *KRAS* G12 mutations in normal tissue is predicted to have a huge mismatch with that observed in CRC, indicative of a high potential for augmenting transformation. On the other hand, while *KRAS* mutations Q61L and A146P did display a mismatch, the relative size of this was small, suggesting (within the limited statistical margins we can derive from the credible intervals) that there is little potential for these mutations to augment carcinogenesis. We note the ambiguity in assigning Q61L and A146P as CRC drivers but consider that this is beyond the scope of the current study and include it here solely for the benefit of the Reviewer. We thank them again for raising this interesting question that provides further support to our subsequent focus on KRAD G12 mutations in terms of their relevance for CRC.

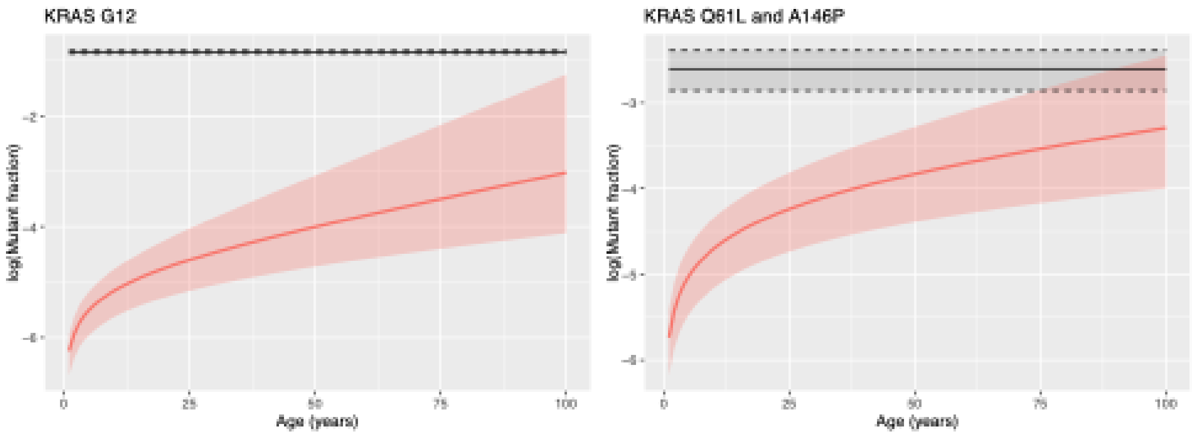

Figure R2. Predicted mutational burden curves as a function of age for the two groups of *KRAS* missense driver mutations in normal tissue (red lines with 95% credible interval values) compared with observed frequency of the same mutations in CRC (black lines with 95% binomial confidence interval). Intersection of the two lines over the course of a lifetime is interpreted as evidence for the selection dynamics of those mutations in normal tissue being sufficient to explain their occurrence in CRC.

KRAS mutations were called in 80% of the REG4-high regions. Were these regions also analyzed for mutations in NRAS or BRAF to explain the other 20%?

This is a very insightful comment. Indeed, there have been reports where serrated lesions with *BRAF* mutations are associated with a REG4 high transcriptomic signature (Chen *et al.*, 2021). We had previously performed next generation sequencing for *BRAF* V600E for the 20% of REG4 high regions that did not have a KRAS mutation and did not find any mutations. We have now included this in the manuscript (lines 338-341 and Figure 4f in the revised manuscript).

For a more complete response, we have now performed Sanger sequencing for all the REG4 positive patches for *BRAF* V600E and indeed no mutations were detected (Figure R3).

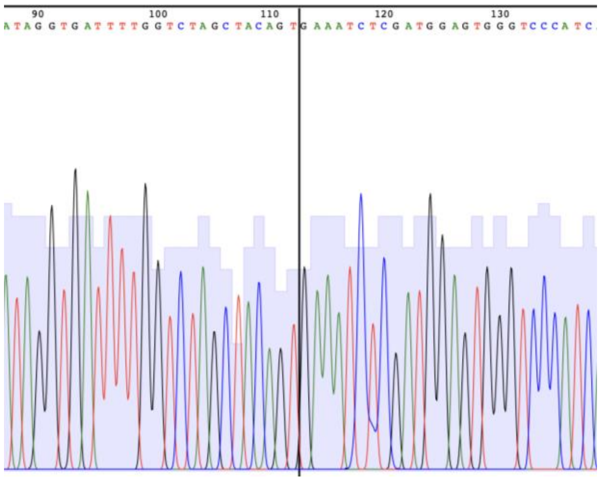

Figure R3. Sanger sequencing trace of REG4 high patch sequenced for BRAF V600E mutation. T>A substitution would be expected if there was a mutation.

Unfortunately, the laser-capture material was limited in terms of quantity and volume so we could not perform any further sequencing for *NRAS*, but we have now included in the manuscript that we cannot exclude this possibility (line 341 in the revised manuscript).

Regarding the spatial transcriptomic dataset. I may understand that subtle phenotypes for heterozygous tumor suppressors is challenging to pick up. Is the potential clone size of FBXW7 sufficient to be picked up, in relation to KRAS mutant clones that expand in size and elevate their relative contribution to the semi-bulk RNA profile?

Yes indeed, we did not detect any transcriptomic changes in samples with *FBXW7* mutations, as we did with *KRAS* mutations. To investigate this further, we went back to our spatial transcriptomics data and looked at the localised areas where the *FBXW7* R465C mutations were detected and plotted the barcodes on a UMAP to look for any deviation of some potential glands, as in the example shown below. We included the barcodes of the larger area where the clone was located and then examined whether in the spatial region that the mutant clone was detected (Figure R4, black box) there were any distinct clusters. In the example in Figure R4, it is evident that distinct clusters can be detected (pink dots, cluster 7) but this corresponded to a histological region of submucosa. The spatial areas where the clones were located did not have any unique clusters.

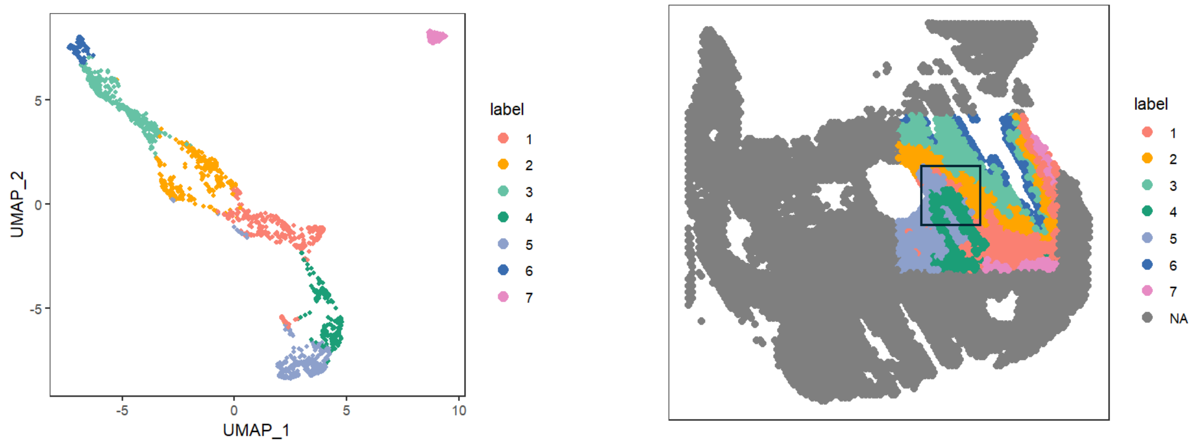

Figure R4. Spatial transcriptomics with a region of tissue carrying *FBXW7* R465C. The analysis was performed on a sub-region of the whole section that contained the *FBXW7* R465C mutation. Left shows UMAP with distinct clusters. Right shows the same in the spatial context of the whole section. Black square indicates the area where FBXW7 R465C clone was located. No unique transcriptomic cluster is seen in the area with the clone.

The reviewer's explanation remains plausible, and we cannot formally distinguish a subtle transcriptional phenotype from one that is disguised due to small clones being masked by a background of normal cells. *KRAS* G12 clones are typically large due

to an elevated fission rate, but the higher fixation bias associated with *FBXW7* R465C clones is expected to increase occurrence while clones remain smaller by. We have updated the revised manuscript to clarify this point (line 319-321 in the revised manuscript).

Reviewer #2 (Remarks to the Author): CRC, spatial transcriptomics, clonal evolution and computational biology

We received the manuscript entitled, “Clonal biases dictate availability of colonic cancer driver mutations for transformation”, with substantial enthusiasm. Our opinion is of that the study addresses an important and timely topic in the field – of the clonal dynamics of driver mutations that exist in phenotypically normal tissues. The authors utilize sequencing technologies on a cohort of human colonic specimens along with computational modeling to differentiate models of clonal expansion (fixation – monoclonal crypt conversion vs. fission resulting in increased patch size). The study was well done and was able to differentiate these two models based on mutational patterns in different oncogenes. The KRAS result has been reported in a previous study (Olpe et al., 2021). There are areas in the paper that the authors should address to enhance clarity.

We thank the Reviewer for their positive perspective on the manuscript.

Comments:
1. The authors utilize 3 resolutions of targeted amplicon sequencing, micro-seq (all the way down to crypt level), aceStrip-seq (tissue strips), and Section-seq (bulk). The authors should clarify how those technologies are used. Right now, it seems like micro-seq is predominantly used.

The three scales of sequencing were originally applied anticipating that the sweet spot for resolution would differ depending on whether frequent small clones or infrequent large clones were present. However, a limitation of using sequencing to obtain information on clone selection and dynamics is that multiple combinations of clone numbers and sizes are compatible with a reported VAF value, as described in the original manuscript. Because of this, we primarily used Micro-seq data (the smallest scale sampled) to quantify selection biases based on the smaller clone sizes it can detect. We then were able to use the other datasets at larger scales (AceStrip-seq and Section-seq) to 1) validate list of mutations called and 2) independently assess predictions of mutational burden based on inferred clone dynamics. In the revised version of the manuscript, we have attempted to make this original complementary use of the different scales more apparent for the reader (lines 113-125 and 163-165 in the revised manuscript).

In addition, we have now included some additional analysis of AceStrip-seq data based on sequencing serial sub-sections from samples with certain driver mutation

calls to address the issue of driver co-occurrence (using sub-AceStrip-seq, see response to this Reviewer's point on ordering below). In summary, this provides a third use of other scales beyond Micro-seq that we have appropriately described within the revised version of the manuscript.

Finally, to increase the sample size for the spatial transcriptomics, mutant samples were used from both Micro-seq and AceStrip-seq, where the spatial location of clones is accurately noted. This is now highlighted in the Methods (line 831-832).

2. For micro-seq, the authors should also clarify if single crypts are sequenced like previous studies. For some of the results, it seems like multiple crypts are grouped together and sequenced.

We are sorry for the confusion. Indeed, in this study we did not sequence individual glands and around 200 crypts would have been grouped together. This approach allowed us to screen almost an 80-fold higher level of glands as previous reports and we believe is the reason we were able to detect infrequent events, such as truncating APC mutations. We have updated the revised version of the manuscript to clarify this point (lines 104-106, 155-157 in the revised manuscript).

3. As above only 285/76800 clones were determined to have mutations, yet ~41% APC mutations and 18% KRAS mutations were detected. These drivers were not detected in previous studies; please reconcile these discrepancies (i.e. single vs multi crypts sequenced for example).

We thank the reviewer for giving us the opportunity to argue the merits of our approach. To avoid confusion, we note that the reported "~41% APC" and "~18% KRAS" values refer to the fraction of the 285 clones in which any mutation was detected, not to the fraction of all crypts screened. The 76,800 refers to the total number of crypts that we estimated were captured in in the 384 LCM samples each of which contained around 200 crypts. The optimal choices between sample size (as per the Reviewer's point 1 above) and sequencing modality and depth were foundational in the study design. In effect, targeting a small number of relevant events across larger areas of tissue made the study feasible. We believe this explains why we were successful in detecting driver events that were missed by other studies that sequenced many targets in very small areas (one crypt) areas of tissue. Taking APC truncating mutations as an example, we detected just 11 of these among the 76,800 crypts screened with our amplicon panel, designed to capture most known cancer driver mutations in this gene. These values provide an estimated occurrence for APC truncating mutations of 1.43 per 10,000 crypts (0.0143% of crypts, standard error 0.004318%). Lee-Six et al sequenced 2,035 individual crypts, either by whole genome sequencing or using a targeted exome panel, both with the possibility to detect these same mutations. Based on a binomial model using these values, the probability of finding at least one truncating APC mutation is just 25.28%.

Stated another way, the expected number of truncating APC mutations one would find among 2,035 based on these values is 0.29, consistent with none being reported by the Lee-Six et al study. We have now provided a justification for identifying these mutations in the main text (lines 155-157 in the revised manuscript).

4. Most of the images provided were tissue sections of colonic crypts en face. Can you comment on the artifacts produced given the crypts are not sampled cross-sectionally. I.e. especially for the fixation model, that you are not sampled the top and bottom of the crypts entirely?

The Reviewer is correct that we sampled *en face*. This has the advantage that circular crypt areas within the 200-crypt sample are relatively consistent compared to crypts cross-sectioned longitudinally. We have estimated that the minimum mutant size we can detect corresponds to a single mutant crypt. Rather than causing artefacts we believe that there is a limitation to the method in that the sample size dictates that most clones that are only partially populating the crypt are below the detection threshold for the method. Notably the inferences of clone dynamics derived here do not require information on the presence or size distribution of such small intragland clones.

5. A limitation in the study is that mutations in only 5 genes were profiled. The mutation statuses of many genes are unknown, and it is very likely that there are interactions amongst mutations that give rise to phenotypes that are missed in the study. A point I would like to make about this is the phenotypic heterogeneity conferred by KRAS mutations (Fig. 4h), which may have arisen due to other mutations occurring in the tissue. Specifically, what about the ORDER of mutations between Apc-KrasG12 compared to KrasG12-Apc? Do these scenarios give different phenotypes, such as one giving rise to the hyperplastic polyp phenotype? The order can probably be deciphered by looking at VAF differences between the two mutations.

We agree with the Reviewer that one limitation of our study is the small region of the genome covered, compared to studies from e.g. the Sanger Institute where either whole exome or genome sequencing of single crypts has the potential to detect more mutations per crypt. However, we point out that our aim was to provide a complementary dataset where targeted sequencing of smaller regions at higher depth would allow more sensitive detection of mutated crypts within larger regions of tissue (see responses to this Reviewer's points 1 and 2 above). Thus, the amplicon panel was specifically designed to query the prevalence of and interaction between the "key" CRC driver mutations in normal tissue with the trade-off of potentially missing other mutations that may be co-occurring within the tissue.

We highlight that very few instances of co-occurrence of the targeted CRC driver mutations were detected within the same Micro-seq sample (just five out of 384 samples, as now reported in the main text lines 249-253/Suppl. Table 6). Moreover,

in each case, the VAFs of both mutations were too low to make a shared clonal origin probable ($p < 0.0001$ assuming two or fewer crypts harbour either mutation). Given the relevance for CRC as highlighted by both Reviewers, in the revised manuscript we therefore incorporated a new experimental dataset (sub-AceStrip-seq) in a new results section specifically designed to identify instances of driver co-occurrence and potentially ordering. Briefly, we selected cases where single driver mutations were called at high VAF values at AceStrip-seq level and further sub-sliced and sequenced tissue serial to these locations with the intention of identifying large patches or “fields” of mutated crypts. With an understanding that the majority of the crypts in such sub-AceStrip-seq would contain the major driver mutation, we rationalised that any other driver mutations called within those same samples at lower VAF (and perhaps initially undetectable at AceStrip-seq level) would necessarily correspond to crypts harbouring both mutations. As presented in the revised version of the manuscript, the new approach did not identify any instance of driver co-occurrence, suggesting these events are incredibly rare. We thank both Reviewers for motivating this new analysis. These new results are presented on lines 246-263 and Fig. 3c of the revised manuscript.

Given the paucity of co-occurrent driver events observed in our data, we instead opted for a simulation-based approach to more thoroughly address the Reviewer’s comment on mutational ordering. As described in response to Reviewer #1’s point previously, we adapted an established simulation model that was used by the original authors to identify the most common ordering of events for initiation of CRC (assuming biallelic inactivation of APC and TP53, and monoallelic activation of KRAS) (Paterson *et al*, 2020). Applied to our scenario, the model adapted with our own inferred clone dynamics parameters was used to study the emergence of crypts with a triplet of mutations in three different genes, which provides a refinement of the results reported in the original modelling paper. In response to Reviewer #1, we have also included the possibility that biallelic inactivation of APC leads to a high selection bias. As we describe in the revised manuscript, the most probable ordering of events that results in a triplet mutated crypt is governed by an interplay between the *de novo* mutation rates and relative selection biases attributed to each mutated gene. When a large fixation and fission bias are assumed for biallelic APC inactivation, by far the most probable route to a crypt with a mutation in all three genes which emerges in older patients (age 80) is one that first acquired biallelic inactivation in APC. However, at younger age (age 50), crypts where a *KRAS* G12 driver mutation occurred first are equally likely as those with an APC inactivating mutation (probability of KRAS as a first founder event is 42.9% versus 4.7% of clones at ages 50 versus 80, respectively). This analysis has added a different context to our work, and we thank the reviewer for motivating it. It can be found in lines 289-306 and Fig. 3e in the revised manuscript.

6. The nuisances and assumptions of the computational model are also quite limited (for example, assuming independence of mutations and independence of

monoclonal conversion and fission). It would be helpful to biologists to explain the model better in terms of how the two models are independent (one would that both crypt fission and fixation depends on biased proliferation). Also, it would seem like fixation is a rather limited form of clonal expansion since it will only be limited to a single crypt, while fission will generate patches of monoclonal crypts. How the model of clonal dynamics from individual patients is defined and related to “lifetime risk of developing cancer” is also not clear.

We apologise for not being able to fully explain the model employed in the previous version of the manuscript. As described in responses to both Reviewer’s points above, in the revised manuscript we have we instead presented our simulations results using an established mathematical model (Paterson *et al*, 2020), which simultaneously enabled us to study the impact of biallelic APC inactivation, comparison with CRC incidence rates and mutational ordering, all within one single framework. In the new results and methods sections associated with these results (lines 268-275 and 774-792 in the revised manuscript), we explain how the model incorporates clone expansion via fixation and fission. We also hope that by using this already published, highly cited mathematical model for CRC initiation, any nuances and/or assumptions are already familiar or at least immediately accessible to researchers within the field, and we have attempted to clarify those most relevant to the current study in a self-contained fashion within the revised manuscript.

7. The manuscript focuses a lot on KRAS mutations and not so much on FBXW7. This is due to regions with KRAS mutations exhibiting very different transcriptomic phenotypes compared to the rest of the conditions. However, KRAS mutation confers secretory differentiation, and thus those cells become distinct from colonocytes. This aspect of RAS-MEK-ERK signaling has been known historically (Heuberger et al., 2014). We wonder if this substantial difference in differentiation is masking more subtle effects of the other mutations (such as haploinsufficient effects of loss of 1 allele of APC)

We thank the reviewer for this insightful contribution. Indeed, some of the top markers of the *KRAS* signature (highest fold change) include goblet cell markers, such as *MUC2* and *SPINK4*, which would be in line with a secretory signature. C8 of MSigDb contains colonic secretory cell types, which were not found to be enriched in our original GSEA analysis. To specifically address this question, we resorted to the literature and compiled our own Goblet cell signature. GSEA analysis was repeated with this gene set but there was no significant enrichment. So, in our hands, *KRAS* mutations in normal tissue seem to confer a more fetal/metaplastic rather than traditional secretory/goblet cell phenotype. We have mentioned this in the revised manuscript (lines 365-366 in the revised manuscript).

The reviewer is correct about the fact that because of the strong transcriptomic phenotype of *KRAS* mutations, the effect of other mutations with less profound

changes could be masked. However, the ability of *KRAS* mutations to mask the effects of other mutations would seem to apply to contexts where these co-occur. As stated above, we believe the events we detect are largely independent of each other.

8. Are biallelic loss of APC function detected in “normal” colon?

As described in response to Reviewer #1’s point on the same topic, we did not detect any biallelic *APC* mutations in normal colon and have therefore made sure to highlight that all reported events are assumed monoallelic in the revised manuscript. Please see the response to Reviewer #1 for our more detailed treatment of biallelic APC inactivation.

9. From Simon Leedlam’s work, there are defined signatures for stem-like tumor cells and fetal-like tumor cells (or adenoma vs serrated cells). This current paper tarnscriptomically examined the fetal/serrated/secretory signatures. We are wondering if the authors can also query the stem signatures.

This is a very good point, thank you. We did indeed consider stem cell signatures but did not find any enrichment in them. We have mentioned this in the revised manuscript as it is indeed an important distinction (line 365-366 in the revised manuscript).

10. Regarding lineage confusion, one really needs to demonstrate that cells are lineage confused at the single cell level (i.e. both signatures admixed in one cell). Right now, it seems like a region is lineage confused but the signatures are expressed in different parts of the region (in different cells). Also, even if there are cells that expressed mixed signatures, this may be a transitory state (one state on its way of converting into another state) and not a permanent mixed identity, and thus, cannot be formally defined as lineage confusion.

We thank the reviewer for highlighting this important point. We have now performed immunofluorescence of previously defined lineage confused clones to obtain the single-cell resolution lacking in the Visium data (Fig. 4k and lines 372-374 in the revised manuscript). We find evidence of some cells expressing both gastric (MCU5AC) and intestinal (CDX2) markers simultaneously, but not all cells of a gland have that mixed phenotype. It is true that we cannot fully exclude the possibility that this is a transitional phenotypic state rather than a permanent feature of being trapped between lineages and have therefore changed our terminology from lineage confused to lineage mixed when referring to these cells in the revised manuscript (lines 374-378 in the revised manuscript).

Minor:
Figure 4E needs a randomized control.

Thank you for highlighting this point. The association between *KRAS* missense mutations and REG4 expression has now been assessed using Fisher's exact test, applied to the contingency data from paired REG4-IHC and *KRAS* sequencing of REG positive/negative regions of tissue not previously analysed by Micro-seq (our interpretation of what the reviewer means by a "randomised control" in this case). The rejection of the null hypothesis of no association between REG4 staining and *KRAS* mutation was strongly favoured (two-sided p = 0.00699, one-sided p = 0.00350 testing enrichment of *KRAS* in REG4 positive regions). This has been added to the revised manuscript (lines 331-338) and the results are now presented in Fig. 4f.

Response to reviewers references

Cagan A, Baez-Ortega A, Brzozowska N, Abascal F, Coorens THH, Sanders MA, Lawson ARJ, Harvey LMR, Bhosle S, Jones D, *et al* (2022) Somatic mutation rates scale with lifespan across mammals. *Nature* 604: 517–524

Cheek D, Blohmer M, Nowak M, Antal T & Naxerova K (2025) Age distinguishes selection from causation in cancer genomes. doi:10.1101/2025.10.06.680730 [PREPRINT]

Fischer JM, Schepers AG, Clevers H, Shibata D & Liskay RM (2014) Occult progression by Apc-deficient intestinal crypts as a target for chemoprevention. *Carcinogenesis* 35: 237–246

Gaynor L, Singh H, Tie G, Badarinath K, Madha S, Mancini A, Bhattacharya S, Hoshino M, de Sauvage FJ, Murata K, *et al* (2025) Crypt density and recruited enhancers underlie intestinal tumour initiation. *Nature* 640: 231–239

Gerstung M, Jolly C, Leshchiner I, Dentro SC, Gonzalez S, Rosebrock D, Mitchell TJ, Rubanova Y, Anur P, Yu K, *et al* (2020) The evolutionary history of 2,658 cancers. *Nature* 578: 122–128

Lawson ARJ, Abascal F, Nicola PA, Lensing S V., Roberts AL, Kalantzis G, Baez-Ortega A, Brzozowska N, El-Sayed Moustafa JS, Vaitkute D, *et al* (2025) Somatic mutation and selection at population scale. *Nature*

Lee-Six H, Olafsson S, Ellis P, Osborne RJ, Sanders MA, Moore L, Georgakopoulos N, Torrente F, Noorani A, Goddard M, *et al* (2019) The landscape of somatic mutation in normal colorectal epithelial cells. *Nature* 574: 532–537

Martincorena I, Raine KM, Gerstung M, Dawson KJ, Haase K, Van Loo P, Davies H, Stratton MR & Campbell PJ (2017) Universal Patterns of Selection in Cancer and Somatic Tissues. *Cell* 171: 1029-1041.e21

Paterson C, Clevers H & Bozic I (2020) Mathematical model of colorectal cancer initiation. *Proceedings of the National Academy of Sciences* 117: 20681–20688

Skoufou-Papoutsaki N, Adler S, Mehmed S, Tume C, Olpe C, Morrissey E, Kemp R, Girard A-C, Moutin EB, Chilamakuri CSR, *et al* (2025) Haploinsufficient phenotypes promote selection of PTEN and ARID1A-deficient clones in human colon. *EMBO Rep* 26: 1269–1289

Sottoriva A, Kang H, Ma Z, Graham TA, Salomon MP, Zhao J, Marjoram P, Siegmund K, Press MF, Shibata D, *et al* (2015) A Big Bang model of human colorectal tumor growth. *Nat Genet* 47: 209–216

Stratton M, Leung SY, Wang Y, Robinson P, Thomas L, West H, Sampson J, Jung H, Fitzgerald S, Lee-Six H, *et al* (2025) The earliest stages of neoplastic transformation in Familial Adenomatous Polyposis. doi:10.21203/rs.3.rs-6581155/v1 [PREPRINT]
